# Bioengineered algal lipids enriched in structured medium- and long-chain triacylglycerols, linoleate, and *sn*-2 palmitate for human milk fat substitutes

**DOI:** 10.64898/2026.05.15.725531

**Published:** 2026-05-16

**Authors:** Jon Y-T. Lin, Marco A. Dueñas, Suzanne Kosina, Anthony T. Iavarone, Kenzo Khoo, Carrie D. Nicora, Samuel O. Purvine, Trent R. Northen, Jeffrey L. Moseley, Sabeeha S. Merchant

**Affiliations:** 1 California Institute for Quantitative Biosciences, University of California, Berkeley, Berkeley, CA 94720, USA; 2 Department of Plant and Microbial Biology, University of California, Berkeley, Berkeley, CA 94720, USA; 3 Department of Molecular and Cell Biology, University of California, Berkeley, Berkeley, CA 94720, USA; 4 Biological Sciences Division, Pacific Northwest National Laboratory, United States Department of Energy, Richland, WA 99352, USA; 5 Environmental Molecular Sciences Laboratory, Pacific Northwest National Laboratory, United States Department of Energy, Richland, WA 99352, USA; 6 Environmental Genomics and Systems Biology, Lawrence Berkeley National Laboratory Berkeley, Berkeley, CA 94720, USA; 7 Department of Chemistry, University of California, Berkeley, Berkeley, CA 94720, USA; 8 Joint Genome Institute, Lawrence Berkeley National Laboratory, Berkeley, CA 94720, USA

## Abstract

Human milk fat (HMF) contains triacylglycerol (TAG) as its primary component, providing over 50% of the calories for infant nutrition, along with structural and bioactive lipids that are important for immune and nervous system development. Palmitic acid, comprising 20–25% of the fatty acid complement of HMF, is predominantly esterified to the *sn*-2 position on the glycerol backbone. This regiospecific positioning facilitates absorption as 2-palmitoyl-monoacylglycerol after hydrolysis of the fatty acids at *sn*-1 and *sn*-2 by gut lipases. Other features of HMF include enrichment in structured medium- and long-chain triglycerides (MLCTs), and variation in the ratio of oleic acid to linoleic acid with maternal diet and geography. We have engineered *Auxenochlorella*, an oleaginous green alga, for biosynthesis of an MLCT- and *sn*-2 palmitate-enriched HMF substitute for infant formula, matching the regioisomeric composition and proportions of the most abundant fatty acids in HMF.

## Introduction

Human milk is an incredibly complex biofluid that provides infants with all necessary nutrition for up to six months postpartum ^1^. Half of the energy for growth is delivered by fat, which is composed primarily of triacylglycerols (TAGs) for calories, but also includes bioactive and structural phospholipids, sphingolipids, sterols and fatty acids with signaling and developmental roles ^2^. The regioisomeric positioning of acyl chains in TAG is important for infant lipid assimilation, with 70–88% of palmitic acid (C16:0), the major long-chain saturated fatty acid in human milkfat (HMF), esterified at the *sn*-2 position on the glycerol backbone ^3^. Saturated, medium-chain fatty acids (MCFA, C8:0–C14:0) or unsaturated, long-chain fatty acids hydrolyzed from the TAG *sn*-1 and *sn*-3 positions by gastric and intestinal lipases are readily absorbed by enterocytes ^4,5^, along with the remaining 2-palmitoyl glycerol ^6^. In contrast, vegetable oil blends commonly used as lipid sources in infant formula are typically enriched in unsaturated long-chain fatty acids at *sn*-2, while palmitate is preferentially esterified at *sn*-1 or *sn*-3 ^7^. The gastric and intestinal lipases thus release palmitic acid, which being less soluble forms non-absorbable soaps with calcium ions, leading to intestinal distress, reduced caloric intake, and calcium deficiency in extreme cases ^8,9^.

Another defining characteristic of HMF is the substantial proportion of MCFA (10–30% of total FA) ^10,11^ and linoleate (C18:2 n-6) (15–25% of total FA) ^10,12^, the levels of which are shaped by maternal diet and geography. Linoleic acid and α-linolenic acid (C18:3 n-3) are essential fatty acids for mammals ^13–15^, and U.S. regulations require linoleate supplementation in formulae ^16,17^. MCFA are a key energy source due to their direct transport from enterocytes to the liver via the hepatic portal vein ^4,18^, as well as bypass of the carnitine shuttle for import into mitochondria for oxidation ^19,20^. MCFA in plant-based formulas are mainly derived from coconut or palm kernel oils enriched with trisaturated medium-chain TAG, in contrast with the structured medium- and long-chain triglycerides (MLCTs) found in HMF ^21^. Clinical data have shown that the latter provides improved health benefits to infants through as yet unclear mechanisms ^21–23^.

HMF substitutes with high *sn*-2 palmitate for infant formula are presently made by enzymatic interesterification from fractionated lipids and free fatty acids derived from chemical hydrolysis or lipase treatment of natural oils ^24,25^. Metabolic engineering for biosynthesis of *sn*-2 palmitate-enriched TAG was first demonstrated in *Arabidopsis* by expressing a version of *Brassica napus* LPAT1, a plastid palmitoyl-ACP-specific lysophosphatidic acid acyltransferase (LPAAT), lacking its plastid transit peptide ^26^. The truncated enzyme was relocated to the endoplasmic reticulum (ER), enabling it to participate in the cytoplasmic Kennedy pathway for glycerolipid biosynthesis ^26^. C16:0 incorporation at *sn*-2 was enhanced further by reducing competition with the endogenous ER LPAAT (LPAT2), and by eliminating acyl-editing catalyzed by phosphatidylcholine:diacylglycerol cholinephosphotransferase 1 (PDCT1) ^26^. A follow-up study achieved 80% C16:0 esterification at *sn*-2 with a human palmitoyl-CoA-specific LPAAT (AGPAT1) ^27^ in a genetic background optimized for increased C16:0 abundance and inhibition of competing pathways ^28^. These findings demonstrate the feasibility of engineering oilseed crops to accumulate *sn*-2 palmitate, but the approach is constrained by slow development cycles and regulatory bottlenecks, ultimately exacerbates the environmental impact of land-use conversion for agriculture.-.

Heterotrophic cultivation of oleaginous microorganisms offers another route for producing HMF substitutes. *Chlamydomonas reinhardtii* palmitoyl-CoA-specific LPAAT2 ^29^ was introduced into an *obese* strain of the oleaginous yeast, *Yarrowia lipolytica* ^30^, and yielded TAG with 62% of C16:0 at *sn*-2 when grown on a mixture of glucose and palm oil ^31^. Similarly, *Y. lipolytica* strains expressing human AGPAT1 or *C. reinhardtii* LPAAT2 were reported to accumulate 18–25% of their TAG as 1,3-dioleoyl-2-palmitoyl glycerol (OPO) ^32^. Zhao et al (2023) carried out an extensive screen to assess the C16:0 selectivity of 28 different LPAATs, mostly from green algae, in *Prototheca wickerhamii*, an oleaginous, non-photosynthetic trebouxiophyte alga ^33^. C16:0-selectivity was improved in strains expressing LPAATs from *Pedinophyceae* sp. and *Edaphochlamys debaryana* compared to the human and *C. reinhardtii* enzymes ^33^. Introduction of an unspecified LPAAT from this screen into a classically improved *Prototheca* strain with increased palmitic acid accumulation resulted in lipids with up to 38% OPO, 73% of C16:0 at *sn*-2, and lipid titer of over 150 g/L in a five day fermentation ^33,34^. These outcomes demonstrate commercial metrics for microbial production of engineered *sn*-2 palmitate lipids.

*Auxenochlorella* spp. are emerging reference organisms for discovery biology and biotechnology ^35,36^. Closely related to *Prototheca* and similar in their capacity for heterotrophic growth ^37,38^, transformation of the nuclear genome via homologous recombination ^39^, and triacylglycerol biosynthesis triggered by carbon:nitrogen imbalance (Fig. S1) ^40^, *Auxenochlorella* spp. offer advantages with respect to pigment and cofactor biosynthesis as production platforms for designer lipids. In this study, we build on prior *sn*-2 palmitate bioengineering efforts and describe systematic, multi-step engineering of the hybrid strain, *Auxenochlorella protothecoides x symbiontica* UTEX 250-A (hereafter referred to as *Auxenochlorella*). This approach yielded single source lipids that replicate the abundances of MCFA and linoleic acid, as well as the *sn*-2 palmitate and MLCT regioisomeric composition characteristic of HMF.

## Results

### Engineering *Auxenochlorella* lipids to match the major fatty acid composition of HMF

**Figure 1.**
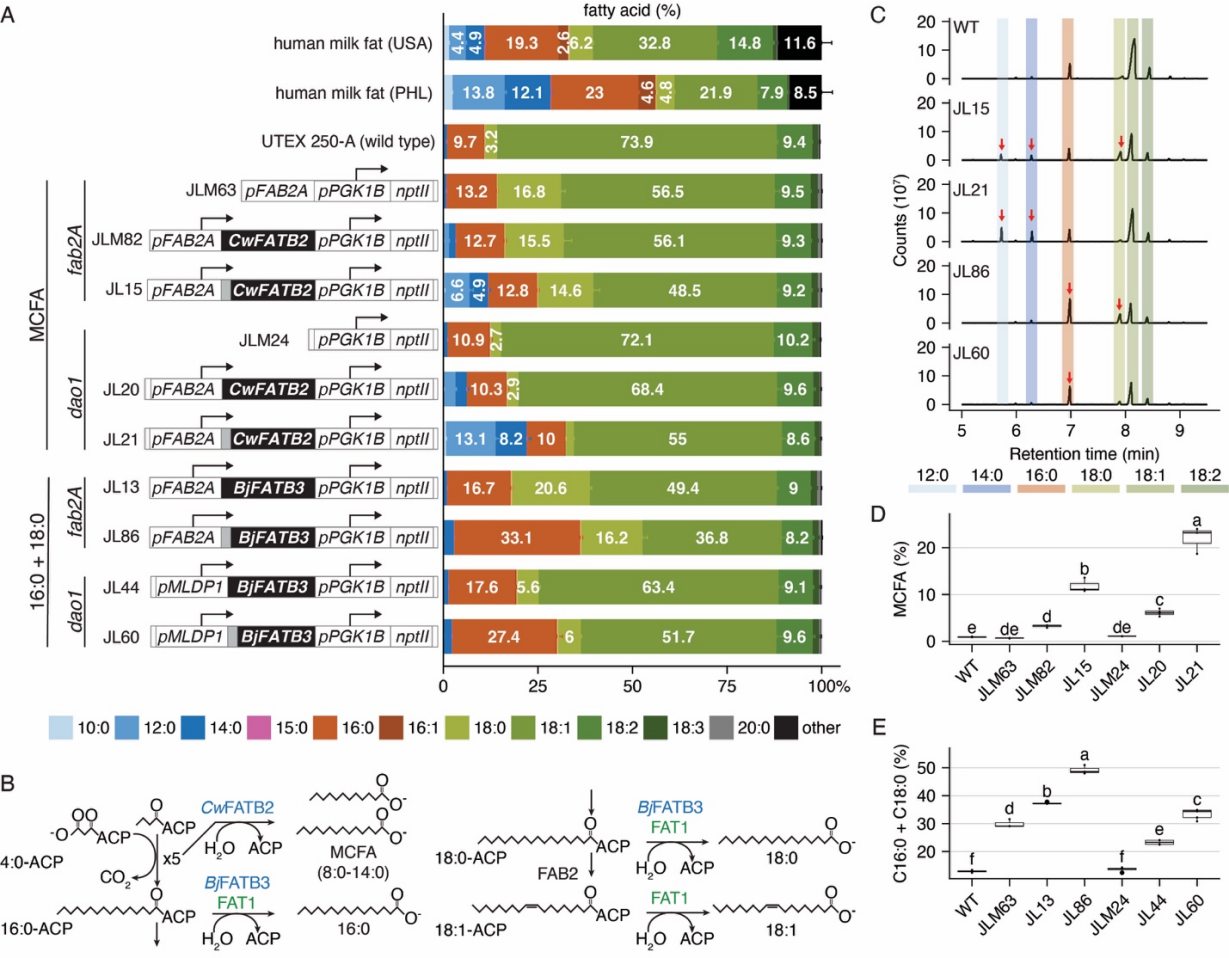
Heterologous thioesterases modify *Auxenochlorella* fatty acid chain length **(A)** Fatty acid compositions (% of total fatty acids) of HMF from the United States (USA), Philippines (PHL) ^3,10^, and heterotrophically grown *Auxenochlorella* wild type and engineered strains in this study. Schematics of the expression constructs are shown on the left. Constructs contain 5’ and 3’ DNA sequences targeting integration at the *FAB2A-2* (JLM63, JLM82, JL15, JL13, JL86) or *DAO1* (JLM24, JL20, JL21, JL44, JL60) loci by homologous recombination. Arrows represent the transcription start sites within promoters (*pFAB2A*, *pMLDP1*, *pPGK1B*) controlling downstream genes. *FAB2A* encodes stearoyl-ACP desaturase 2A, *MLDP1* encodes major lipid droplet protein 1, and *PGK1B* encodes phosphoglycerate kinase 1B. The *nptII* selection marker provides resistance to geneticin (G418). A sequence encoding the FAB2A-1 plastid transit peptide (grey) replaces the predicted CwFATB2 and BjFATB3 transit peptide-encoding regions in JL15, JL21, JL86, and JL60. **(B)** Fatty acid chain length engineering in *Auxenochlorella*. The endogenous FAT1 thioesterase is in green text. Heterologous thioesterases are in blue text. **(C)** Gas Chromatography-Mass Spectrometry (GC-MS) analysis of fatty acid methyl esters (FAMEs) prepared from wild-type and transgenic strain total lipids. The plots represent MS ion intensity versus GC retention time (min). Arrows indicate fatty acids that are significantly increased in abundance in the engineered strains compared to wild type. **(D)** MCFA (C8–14:0) levels in strains expressing CwFATB2. **(E)** C16:0 plus C18:0 levels in strains expressing BjFATB3. n = 3–4 independent transformants of each transgenic strain. Refer to Table S1 for the strain details. n = 10 for the UTEX 250-A wild-type control. Error bars = standard deviation. Letters represent Tukey Honest Significant Differences between strains (padj ≥0.05 for measurements with the same letter).

*Auxenochlorella* storage lipid fatty acid profiles are simpler than those of HMF, with oleic acid (C18:1 n-9) as the dominant species, linoleic and α-linolenic acids as the most abundant polyunsaturates, and palmitic, stearic (C18:0) and myristic (C14:0) acids as the major saturated fatty acids (Fig. 1A and S1). Compared to *Auxenochlorella* lipids, HMF has less oleate but more palmitate and MCFA (Fig. 1A). Since fatty acid chain length is dictated by the substrate specificity of thioesterases that cleave the elongating acyl group from acyl carrier protein (ACP), we introduced heterologous plant thioesterases to modify *Auxenochlorella* fatty acid composition (Fig. 1B and Table S1). A codon-optimized gene encoding CwFATB2, a well-characterized fatty acyl-ACP thioesterase from *Cuphea wrightii* with selectivity for C10:0–C14:0 ^41^ was targeted to two distinct loci. *CwFATB2* was integrated at either allele 2 of the *Fatty Acid Biosynthesis 2A* (*FAB2A-2*) locus, encoding stearoyl-ACP desaturase, causing simultaneous elevation of C18:0 at the expense of C18:1 n-9, or at the homozygous *DAO1* locus, encoding D-amino acid oxidase 1, which is neutral with respect to fatty acid biosynthesis (Fig. 1A, C–D). Lauric (C12:0) and myristic acids were the predominant MFCA in the modified strains, ranging between 3–21% of total fatty acids. The highest accumulation occurred in strains JL15 and JL21, in which the plant plastid-targeting transit peptide is replaced with the *Auxenochlorella* FAB2A plastid transit peptide (Fig. 1A, C–D) ^42^. Untargeted proteomic analysis indicated that the endogenous plastid targeting sequence confers a 3.5-fold improvement in abundance of the heterologous thioesterase (Table S2), consistent with more efficient import and processing of the foreign protein, conferring increased enzymatic activity. Stearic acid levels increased to 14.6–16.2% in *fab2A-2* mutant strains (Fig. 1A, E), which exceeded the target range. Consequently, we pursued alternative strategies for achieving the HMF C18:0 target (4.8–6.2%, Fig. 1A).

BjFATB3 from *Brassica juncea*, was reported to increase C18:0 content in *E. coli* ^43^. However, when expressed in *Auxenochlorella* with either its native or an algal plastid transit peptide, BjFATB3 increased palmitic acid and stearic acid accumulation (Fig. 1A, C, E, compare strains JL44 and JL60 to JLM24 and wild type), suggesting substrate preference for both C16:0- and C18:0-ACP (Fig. 1B). The highest C16:0 levels were achieved in strains JL86 (33.1%) and JL60 (27.4%), compared to less than 10% in wild type (Fig. 1A, C, E). Stearic acid abundance doubled in strains expressing *BjFATB3* from the neutral *dao1* locus compared to wild type and the JLM24 transformation marker-only control, (Fig. 1A, C, E), bringing C18:0 into the HMF target range. We concluded that the HMF target amounts of C12:0, C14:0, C16:0 and C18:0 could be achieved via co-expression of CwFATB2 and BjFATB3. We also note that the JL13 and JL86 strains, expressing the *B. juncea* thioesterase gene in the *fab2A-2* mutant background, represent an intermediate engineering stage in the production of high-stearate structured fats such as those found in cocoa, mango and shea butters ^44,45^.

### Enhanced C16:0 *sn*-2 regioisomeric positioning with heterologous LPAATs

**Figure 2.**
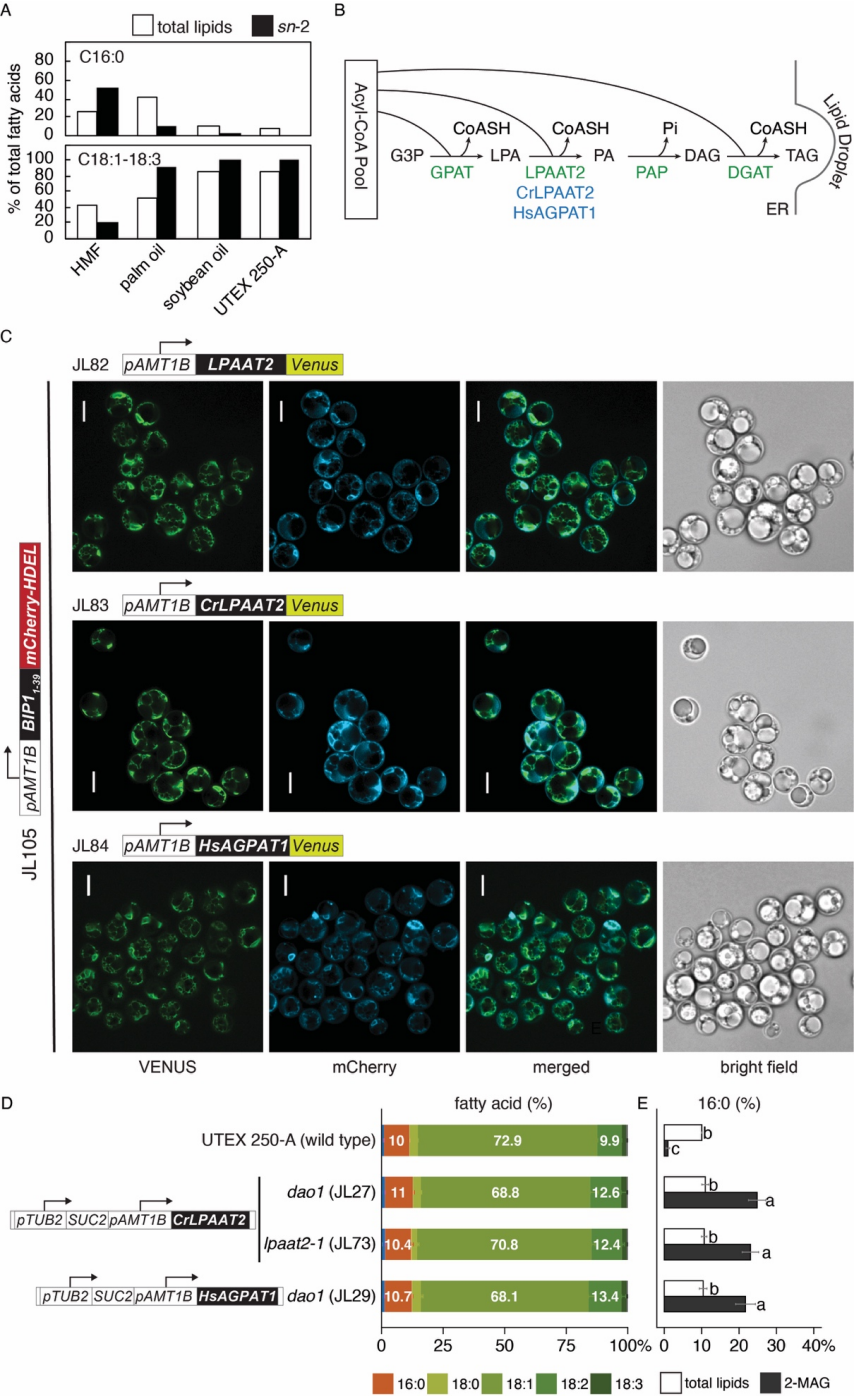
Heterologous ER lysophosphatidic acid acyltransferases enhance positioning of C16:0 at *sn*-2 in TAG. **(A)** C16:0 and unsaturated C18 (C18:1 n-9 + C18:2 n-6 + C18:3 n-3c) proportions of total fatty acids (white bars) and at *sn*-2 (black bars) in HMF ^3^, vegetable oils ^3,92,93^, and in *Auxenochlorella* lipids. **(B)** Kennedy pathway engineering in *Auxenochlorella* for improved *sn*-2 palmitate biosynthesis. Endogenous *Auxenochlorella* enzymes are in green text; introduced genes are in blue text. G3P = glycerol-3-phosphate, LPA = lysophosphatidic acid, PA = phosphatidic acid, DAG = diacylglycerol. GPAT = glycerol-3-phosphate acyltransferase, LPAAT = lysophosphatidic acid acyltransferase, PAP = phosphatidic acid phosphatase, DGAT = diacylglycerol acyltransferase. **(C)** ER co-localization of C-terminal-Venus-fused LPAATs and N-terminal BIP1 signal-peptide fused to mCherry in lipid-producing cells (see Materials and Methods). Scale bar = 5 μm. **(D)** Fatty acid profiles of strains expressing heterologous LPAATs. Only the most abundant fatty acids are shown (>1.5% of total). Error bars = standard deviation. *SUC2* marker gene expression is controlled by the *C. reinhardtii TUB2* promoter (*pTUB2*), and the *Auxenochlorella AMMONIUM TRANSPORTER 1B* allele 1 promoter (*pAMT1B-1*) drives *CrLPAAT2* and *HsAGPAT1* gene transcription. **(E)** C16:0 percentage in total fatty acids (white bars) versus in 2-monoacylglycerol (2-MAG) (black bars) from *sn*-2 lipase assays. Letters indicate statistical groups determined by one-way ANOVA followed by Tukey’s post-hoc test. n = 4 independent transformants for JL27, JL29, and JL73, n = 3 independent cultures for the UTEX 250-A wild-type control. Errors bars = standard deviation.

C16:0 enrichment at the TAG *sn*-2 position is a key feature of HMF (Fig. 2A) ^3^. Conversely, in common vegetable oils and in *Auxenochlorella*, the *sn*-2 position is predominantly esterified with unsaturated fatty acids (Fig. 2A). To alter C16:0 regioisomeric positioning in *Auxenochlorella*, we introduced ER-localized heterologous LPAATs with the desired palmitate substrate specificity (Fig. 2B–D) ^27,29^. The total fatty acid profiles in the resulting strains did not change, with comparable C16:0 levels (10–11%) in the wild type and in strains harboring heterologous *CrLPAAT2* from *Chlamydomonas* (JL27, JL73) or human *HsAGPAT1* (JL29) (Fig. 2D). In contrast, 23–25% of the TAGs in the LPAAT-expressors contained C16:0 at the *sn*-2 position, compared to the wild type with just 0.8–1.4% (Fig. 2D and Table 1). Accordingly, the fraction of total C16:0 at *sn*-2 increased from 2–5% in wild type to 72–75% in JL27 and JL29, within the HMF target range of 70–88% (Table 1). *sn*-2 incorporation of C14:0 and C18:0 increased modestly in the LPAAT-expressing strains, with a stronger effect in JL29 expressing HsAGPAT1, indicating that CrLPAAT2 has greater C16:0 substrate selectivity than does the human enzyme (Table S3).

To better improve incorporation of palmitate at *sn*-2 we sought to reduce isozyme competition from the endogenous ER-LPAAT ortholog ^46^. *Auxenochlorella* LPAAT2 was identified on the basis of orthology to *C. reinhardtii* LPAAT2 (Cre17.g738350) ^36^, with authentic LPAAT activity in vivo (Fig S2) ^29^, and the occurrence of an acyltransferase domain (IPR002123) with conserved active site motifs involved in lysophosphatidic acid substrate binding ^47^. The *Auxenochlorella LPAAT2* locus is likely essential, since we could not generate *lpaat2* double knockouts except in backgrounds that expressed the heterologous CrLPAAT2-Venus and HsAGPAT1-Venus fusions, which also validates the function of the *Auxenochlorella* ortholog (Fig. S3, S5). Indeed, *Auxenochlorella* LPAAT2-Venus shows similar perinuclear localization to CrLPAAT2-Venus and HsAGPAT1-Venus; all of which colocalize with an mCherry ER marker (Fig. 2C and S4), demonstrating accurate targeting of both endogenous and heterologous LPAATs to the site of TAG biosynthesis ^48,49^. Wild type and *lpaat2* single allele knockouts exhibited similar growth (Fig. S3C) and lipid content measured as a fraction of biomass at the end of the time course (Fig. S5), but growth and lipid accumulation were reduced in *lpaat2* double knockout strains carrying the *Chlamydomonas* and human enzymes, suggesting that in single copy the heterologous *LPAAT* genes did not fully compensate for the loss of endogenous LPAAT2 function (Fig. S3 and S5). The fatty acid profiles of the modified strains are unchanged (Fig. 2D, S5). When we compared strains expressing *CrLPAAT2* or *CrLPAAT2-Venus* in the wild-type background to the strains lacking one or both endogenous *LPAAT2* alleles we noted no significant increase in C16:0 incorporation at *sn*-2 (Fig. 2D, compare JL27 to JL73; Table S3, compare JL27 to JL83 and JL83+JL33). From these results we conclude that *Auxenochlorella LPAAT2* encodes the Kennedy pathway LPAAT, and reducing competition from the endogenous isoenzyme does not substantially improve C16:0 *sn*-2 regioselectivity catalyzed by the heterologous LPAATs. CrLPAAT2 was chosen over HsAGPAT1 for further strain development because of its greater C16:0 selectivity.

### Stacking genetic modifications to mimic HMF fatty acid and regioisomeric composition

**Figure 3.**
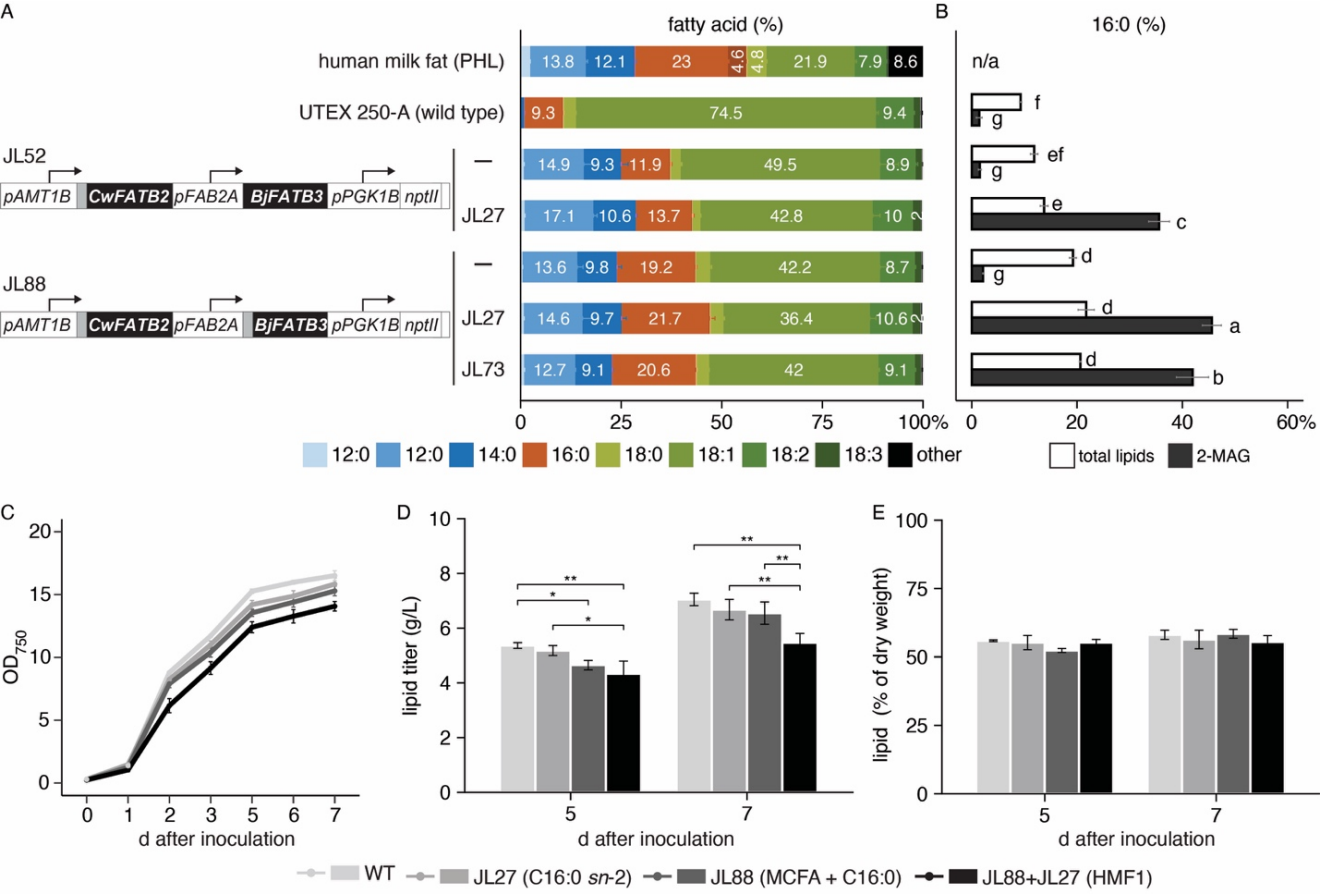
Trait stacking to mimic the medium-chain fatty acids and *sn*-2 palmitate composition of HMF. **(A)** Fatty acid composition comparison of wild-type and engineered strains expressing thioesterases and CrLPAAT2. The thioesterase expression constructs target *CwFATB2* and *BjFATB3* to the *AMT1B-1* allele (3’ flanking DNA not shown). The *AMT1B-1* 5’ flank also serves as the promoter (*pAMT1B-1*) driving the *FAB2Atp-CwFATB2* fusion. The downstream *BjFATB3* gene encoding its native transit peptide (JL52) or fused with the FAB2A-1 transit peptide-encoding sequence (JL88) is driven by *pFAB2A-2*. The expression constructs were transformed into wild-type (—) and the *CrLPAAT2*-expressing strains JL27 and JL73. Error bars = standard deviation. **(B)** Fraction of C16:0 in total FA (white bars) and in 2-MAG (black bars) from *sn*-2 lipase assays. Letters represent Tukey Honest Significant Differences between strains (*padj* ≥ 0.05 for measurements with the same letter). n = 3–4 independent transformants, n = 3 for independent cultures of wild type. Error bars = standard deviation. **(C)** 7-day time course comparing growth of wild type and engineered strains after inoculation in low nitrogen (3.75 mM NH_4_^+^) medium. Tubidity measurements at 750 nm reflect changes in cell number and volume. Refer to Materials and Methods for detailed growth conditions. **(D)** Measurements of lipid titers from 2 mL of cell cultures in C. Error bars = standard deviation. n = 3 independent transformations for each engineered strain and n=3 independent cultures of the wild type. **(E)** Measurements of lipid content as a percentage of dry weight biomass from cell cultures in A. Error bars = standard deviation. n = 3 independent transformations for transgenic strains and n=3 independent cultures of the wild type.

We combined the thioesterase and LPAAT activities to reconstitute the major fatty acid composition and regioisomeric structure of HMF in a single strain. *CwFATB2* and *BjFATB3* were co-expressed in wild type and in strains expressing *CrLPAAT2* at *dao1* (JL27) or at *lpaat2–1* (JL73) (Fig. 3A). Thioesterase-expressing strains JL52 and JL88 produced about 24% MCFA, and 12–22% C16:0 (Fig. 3A), comparable to the saturated fatty acid content of HMF from Filipino mothers (Fig. 1A) ^10,50^. At about 3%, C18:0 was short of the 4–6% target, likely due to competition between endogenous and heterologous thioesterase and β-ketoacyl-ACP synthase activities (Fig. 3A). C16:0 comprises 36–42% of total fatty acids at *sn*-2 in the CrLPAAT2 expressors (JL52+JL27, JL88+JL27 and JL88+JL73) (Fig. 3B, Table 1). With 21–23% MCFA, 20% C16:0, and 68–70% of total C16:0 at the *sn*-2 position, strains JL88+JL27 and JL88+JL73 closely approximate HMF fatty acid content and TAG structure (Fig. 3A, 3B, Table 1). C16:0 incorporation at *sn*-2 was comparable in the stacked trait strain with disrupted *lpaat2–1* (JL88+JL73), compared to the equivalent strain with both *LPAAT2* alleles intact (JL88+JL27), again demonstrating that reducing competition from endogenous LPAAT2 provided no benefit (Fig. 3A, 3B, Table 1). Individually, the modifications to increase *sn*-2 palmitate (Fig. 2D, JL27) and MCFA/C16:0 levels (Fig. 1A, JL88) had only moderate impacts on growth (Fig. 3C) and lipid titer (Fig. 3D). However, when combined in the HMF1 strain, lipid titer decreased by 20–25% compared to the wild type (Fig. 3D, S6, S7). Because the lipid fraction per dry weight remained unchanged across all strains (Fig. 3E), the reduced titer in HMF1 likely stems from slower cell division or a delayed entry into the lipogenic phase.

**Figure 4.**
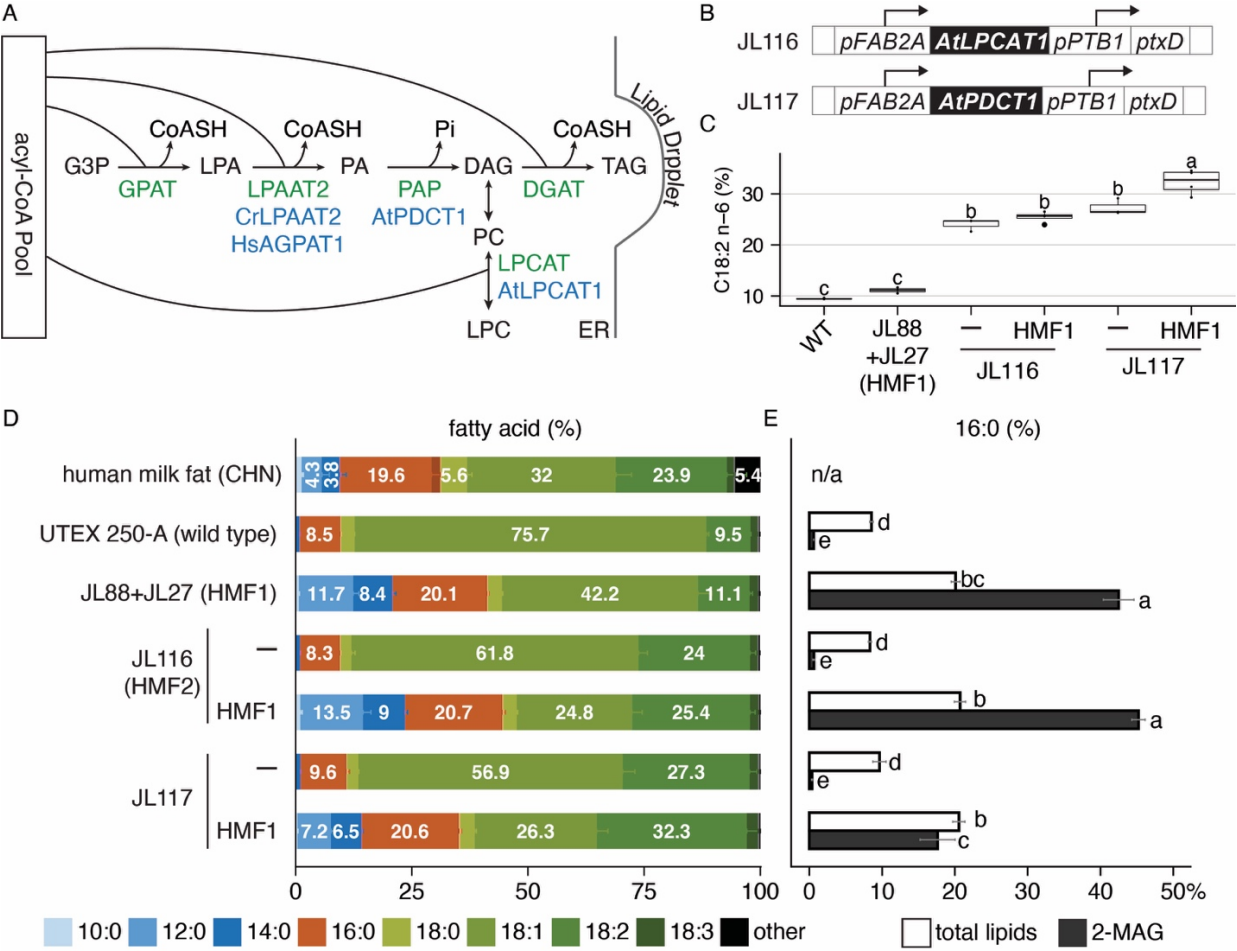
Increased incorporation of linoleic acid into TAG. **(A)** Head-group exchange between phospholipids and DAG by phosphatidylcholine diacylglycerol cholinephosphotransferase 1 (AtPDCT1) and transfer of linoleic acid from phosphatidylcholine (PC) to the acyl-CoA pool by lyso-PC acyltransferase (LPCAT1). LPC = lysophosphatidylcholine. **(B)** *AtLPCAT1* and *AtPDCT1* expression constructs. The *AtLPCAT1* (JL116) and *AtPDCT1* (JL117) constructs contain 5’ and 3’ flanking DNA targeting homologous recombination at the *THI4* locus. **(C)** C18:2 n-6 abundance (% of total fatty acids) in HMF from China (CHN), wild type UTEX 250-A and engineered strains. JL116 and JL117 were transformed into wild-type (—) and the HMF mimic strain JL88+JL27 (HMF1). **(D)** FA profile comparisons of wild type and engineered strains. **(E)** Fraction of C16:0 in total FA (white bars) and in 2-MAG (black bars) from *sn*-2 lipase assays. n = 3–4 independent transformants of each transgenic strain. Error bars = standard deviation. Refer to Table S1 for the strain details. n = 3 for the UTEX 250-A wild-type control. Letters represent Tukey Honest Significant Differences between strains (*padj* ≥0.05 for measurements with the same letter).

Milk from Chinese mothers has the highest linoleic acid levels, averaging over 20% of total fatty acids ^51,52^. *Arabidopsis lysophosphatidylcholine acyltransferase 1* (*LPCAT1*) or *PDCT1* were introduced into wild type or JL88+JL27 (hereafter referred to as HMF1) in order to increase linoleic acid accumulation (Fig. 4A, 4B) ^39,53,54^. Both enzymes increased linoleate abundance two to three-fold at the expense of oleic acid, but LPCAT1 activity did not interfere with C16:0 enrichment at *sn*-2, whereas PDCT1 reduced the fraction of C16:0 at *sn*-2 to 28% (Fig. 4C–E, Table 1 and S3). LPCAT1 is therefore preferred for boosting C18:2 n-6 levels in HMF strains, since it releases *sn*-2 linoleic acid from phospholipids (the site of C18:1 n-9 desaturation) into the acyl-CoA pool for subsequent incorporation into TAG (Fig. 4A) ^53^. In contrast, PDCT1 catalyzes interconversion of phospholipids and diacylglycerol (DAG), and consequently competes with de novo DAG biosynthesis through the Kennedy pathway, resulting in enrichment of unsaturated fatty acids at *sn*-2 (Fig. 4A) ^55^.

### TAG regioisomers in *Auxenochlorella* HMF strains

**Figure 5.**
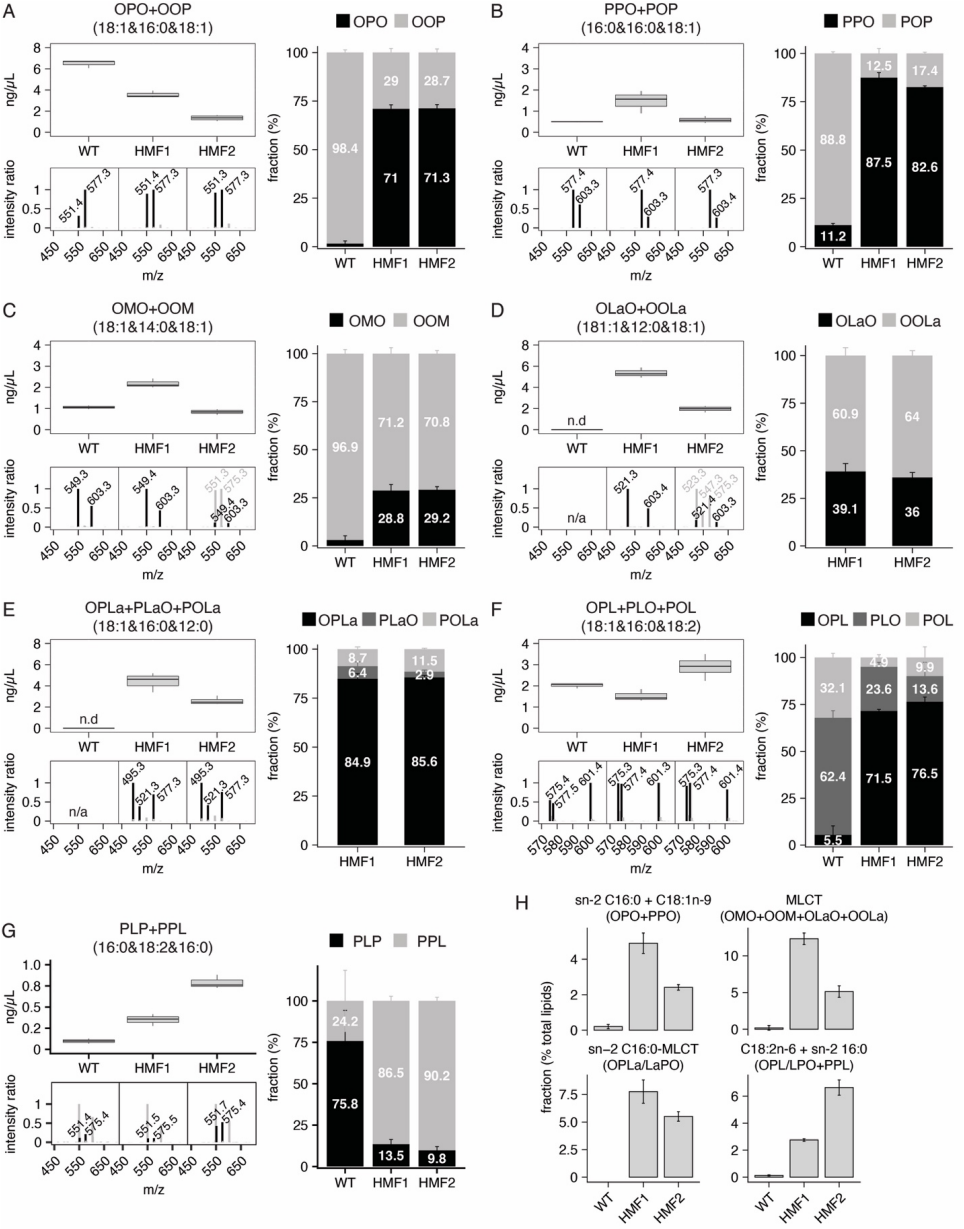
TAG regiospecificity in HMF mimic lipids. (A–G) Seven representative TAGs (total 16 isomers) in total lipid extracts of wild type, JL88+JL27 (HMF1), and JL116+JL88+JL27 (HMF2) were measured and quantified using targeted HPLC-MS/MS. The top-left graph in each panel presents TAG quantification based on MS1 peak height. The bottom-left graphs illustrate spectra that were used to calculate ratios of target MS2 fragments. Regioisomer ratios in the graphs on the right were calculated by comparing sample spectra with spectra from corresponding mixtures of authentic standards for each TAG species. The method does not distinguish between *sn*-1 and *sn*-3 stereoisomers, hence one stereoisomer represents both (e.g. OOP represents both OOP and POO, etc.) (H) Comparison of target TAG regioisomer abundances in wild type, HMF1 and HMF2. The upper left graph shows the proportion of TAGs with *sn*-2 palmitate and oleate (OPO + PPO). The upper right graph presents MLCT (OOM+OMO and OLaO+OOLa). On the lower left, the graph shows quantitation of laurate-containing MLCT with *sn*-2 palmitate (OPLa). Linoleate- and *sn*-2 palmitate-containing TAGs (OPL/LPO + PPL) are presented in the lower right graph. Error bars = SD. n = 3 independent transformations (HMF1) or flask cultures (WT). For HMF2, n= 4 (A-F) or 3 (G and H) independent transformants.

A targeted parallel reaction monitoring (PRM) liquid chromatography-tandem mass spectrometry (LC-MS/MS) method was developed and applied to examine the ratios of key TAG regioisomers in HMF1 and JL116+JL88+JL27 (HMF2) (Fig. 5) ^56,57^. Diacylglycerol (DAG) daughter ion ratios in MS/MS spectra indicate that in wild-type lipid, 98% of dioleoyl-palmitoyl-glycerol molecules were either 1,2-oleoyl-3-palmitoyl-glycerol (OOP), or 1-palmitoyl-2,3-dioleoyl-glycerol (POO), whereas in the HMF mimic lipids 1,3-dioleoyl-2-palmitoyl-glycerol (OPO) comprises 71% of dioleoyl-palmitoyl-glycerol TAGs (Fig. 5A). The absolute abundance of palmitoyl-dioleoyl-glycerol TAGs is highest in the wild type because of the low complexity (mainly oleic, palmitic and linoleic acids) of the fatty acid profile. The engineered strains, designed to have an increased diversity of fatty acids, have lower levels of oleate and hence lower absolute abundance of OPO, OOP and POO (Fig. 5A). Similarly, the *sn*-2 palmitate regioisomers account for 83–87% of dipalmitoyl-oleoyl-glycerols (PPO+POP+OPP) due to increased accumulation of C16:0 in engineered strains over wild type (Fig. 5B). These analyses were broadly consistent with the results of *sn*-2 lipase assays (Fig. 3B, 4E, Table S3)

When we analyzed TAGs containing MCFA, we found that a little over one third of lauryl-dioleoyl-glycerols (OLaO+OOLa) and about 30% of myristoyl-dioleoyl-glycerols (OMO+OOM) have MCFA esterified at *sn*-2 (Fig. 5C, 5D), suggesting that CrLPAAT2 does not discriminate against C12:0 and C14:0. In contrast, 85–86% of lauryl-palmitoyl-oleoyl-glycerols (OPLa+PLaO+POLa) contain palmitate at *sn*-2 (Fig. 5E), reflecting the CrLPAAT2 preference for C16:0. Consistent with the FA profile from GC-MS, laureate-containing TAGs were not detected in wild-type lipid (Fig. 5D, 5E). The predominant regioisomers of palmitoyl-oleoyl-linoleoyl-glycerol (OPL+PLO+POL) and dipalmitoyl-linoleoyl-glycerol TAGs in the engineered lipids also contained *sn*-2 palmitate (72–76% OPL+LPO, Fig. 4F, 86–90% PPL+LPP, Fig. 4G), and as expected, increased linoleic acid in the HMF2 strain expressing LPCAT1 is correlated with higher abundance of OPL/LPO relative to OPO, changing the OPO:OPL/LPO ratio from 2.3 to 0.4. Taken together, LC-MS/MS quantification revealed substantial increases in a broad range of key TAG regioisomers in engineered *Auxenochlorella*, mimicking those in HMF (Fig. 4H).

## Discussion

We have engineered *Auxenochlorella* fatty acid and lipid biosynthesis to mimic the *sn*-2 palmitate, MCFA and linoleic acid content of HMF by stacking genetic modifications in two or three transformation steps, creating a single source algal oil that can be further tailored to match maternal geography or infant nutritional requirements at different developmental stages. Having identified the parts and approach, fully stacked HMF strains may be generated in one or two transformation steps to produce lipids that substitute for vegetable oils in formula (with or without chemical and enzymatic processing). Increased MCFA and C16:0 in TAG were achieved through introduction of select plant thioesterases which terminate acyl chain elongation. Selectivity for palmitate esterification at *sn*-2 was realized using human or *C. reinhardtii* LPAATs. These heterologous LPAATs display ER localization patterns similar to that of the endogenous enzyme and complement the corresponding *Auxenochlorella lpaat2*-null mutant, attesting to equivalent function (Fig. 2C, D).

In strains engineered to accumulate >20% of C16:0, the proportion of palmitate at *sn*-2 was between 68–73%, in line with the 70–88% range of C16:0 at *sn*-2 reported for HMF^3,57,58^. Increased palmitate incorporation at *sn*-2 to reach the upper end of the HMF range can be achieved by introducing highly selective LPAATs from *Pedinophyceae* sp. or *Edaphochlamys debaryana* ^33^. Alternatively, or in concert, heterologous glycerol phosphate acyltransferases (GPAT) and diacylglycerol acyltransferases (DGAT) with low preference for palmitate, such as plastidic GPATs from chilling resistant plants ^59,60^, or DGATs from select plant species ^61–63^ could contribute to exclusion of C16:0 from the *sn*-1 and *sn*-3 positions.

TAGs containing mixed medium- and long-chain fatty acids may be more beneficial for lipid absorption compared to TAGs esterified exclusively with MCFA ^23,64^. Indeed, up to 34% of TAG molecules in HMF contain at least one MCFA ^65^. The engineered *Auxenochlorella* lipids match the relative abundance of medium- and long-chain fatty acids in HMF; notably, lauryl-dioleoyl-glycerols and lauryl-palmitoyl-oleoyl-glycerols are among the most prominent TAG species in both HMF lipid profiles (Fig. 5D, 5E, Table 1) ^65^. Linoleic acid abundance in TAG was increased to match that of oleic acid through the activity of LPCAT1 (Fig. 4C, 4D), resulting in OPO:OPL ratios similar to HMF from Chinese mothers (Fig. 5A, F) ^51^. We could not quantify TAGs containing MCFA and linoleic acid for lack of standards, but we expect lauryl-palmitoyl-linoleoyl-glycerols and myristoyl-palmitoyl-linoleoyl-glycerols to be significant species in HMF2 lipids (Fig. 4D). While there is no evidence for functional differences between HMF with higher OPL versus OPO, consumers may prefer formulae consistent with regional variations in HMF composition.

Looking forward, a realistic objective is to provide a “whole fat” solution for infant formula by engineering single source (clean label) algae oils that fulfill most lipid nutrition requirements, including the recommended levels of very-long-chain unsaturated fatty acids that are important for immune and nervous system development ^2^. The endogenous ER-associated acyl-CoA elongation pathway in *Auxenochlorella* has already been co-opted and augmented with plant and microbial elongases and desaturases to produce arachidonic (C20:4n-6, ARA), eicosapentaenoic (C20:5n-3, EPA) and nervonic (C24:1n-9) acids ^66–68^. One more two-carbon elongation and desaturation step would generate docosahexaenoic acid (C22:6n-3, DHA) from EPA ^69,70^. An added benefit of *Auxenochlorella* lipids is the presence of lutein and zeaxanthin, fat-soluble xanthophylls with benefits for infant eye development and health ^71^. Carotenoid supplementation would likely exceed the recommended dose if all of the fat in infant formula came from *Auxenochlorella* oil ^72^, but manipulation of carotenoid biosynthesis to reduce carotenoid content and change the xanthophyll composition has been demonstrated ^39^. HMF mimic oils could also be engineered to provide vitamin A through expression of β-carotene 15,15'-dioxygenase, retinaldehyde reductase, and acyltransferases for conversion to retinyl esters ^73^. Collectively, the native capacity for lipid and carotenoid accumulation, coupled with tractability towards pathway engineering, position *Auxenochlorella* as a uniquely versatile production platform for lipid ingredients tailored for the complex requirements of infant formula.

## Materials and Methods

### Strains and growth conditions.

All engineered strains (Table S1) were generated in the *Auxenochlorella protothecoides x symbiontica* UTEX 250-A wild-type background, derived from UTEX 250 ^36^. Cells were cultivated heterotrophically in standard ApM1 medium with 2% glucose, 2 μM thiamine-HCl, and 7.5 mM (NH_4_)_2_SO_4_^74^. For lipid-induction, cells were cultured in 12-well plates (Corning^™^ 3512) containing 1 mL of ApM1 with 0.5% glucose (other ingredients unchanged) in the dark, shaken at 150 rpm at 26°C for 3 days in an Innova 44 shaking incubator (New Brunswick), allowing the culture to reach stationary phase. 320 μL of the stationary phase cultures were used to inoculate 4 mL of standard ApM1 and cultures were grown with shaking at 150 rpm at 26°C for 24 h. Aliquots of these cultures were used to dilute cells 50-fold into a total volume of 15–25 mL of ApM1 with reduced nitrogen (3.75 mM (NH_4_)_2_SO_4_) corresponding to a quarter of the nitrogen in the standard medium to promote storage lipid accumulation (day 0). An additional 2% (w/v) glucose was added on day 2 to increase the C/N ratio and increase lipid yield. Samples were collected, unless specified, on d5 during the lipid-accumulating phase for downstream lipid analysis.

### *Auxenochlorella* transformations.

Transformations were performed as described by Dueñas et al. ^75^. Briefly, in 1.5 mL eppendorf tubes, 15 μg (in 10–15 μL) of linearized plasmid DNA was mixed with 150 μL of cells pre-treated with 0.1 M lithium acetate/1X TE pH 8. 750 μL of 40% (v/v) polyethylene glycol-4000 in 0.1 M lithium acetate/1X TE pH 8 was then added, and the mixtures were shaken at 150 rpm in the dark at 26°C for 16 h. Cells were collected by centrifugation at 2348 × *g* for 30 s in an eppendorf 5424 tabletop centrifuge, resuspended in 0.25 mL of 1 M sorbitol, and plated on selective ApM1 agar plates ^74^. Selection markers included 1) *SUC2* for growth on 2% sucrose, 2) *nptII* for selection with 100 μg/mL geneticin (G418 sulfate), 3) *THIC* for selection for growth on thiamine-free media, and 4) *ptxD* for selection for growth on phosphite ^36^. All transformants were analyzed by PCR genotyping and DNA sequencing of the resulting PCR products to confirm integration of the construct at the insertion site. At least three independent transformants from each transformation were chosen for further analysis (Table 1).

### Plasmid construction.

DNA constructs were designed and managed using SnapGene. All heterologous genes, listed in Table 1, were synthesized by GenScript Corporation, USA after codon-optimization to match the codon-bias in UTEX 250-A. DNA fragments were generated by PCR amplification using Herculase Fusion II DNA Polymerase (Agilent 600677) or restriction enzyme digestion of plasmid DNA, then assembled using NEBuilder^®^ HiFi DNA Assembly Master Mix (New England Biolabs E2621L). DNA sequences encoding the predicted *FAB2A-1* (UTEX250_B12.15855.1) gene promoter (*pFAB2A*) and transit peptide (1–38 aa, FAB2Atp, Table 1) were amplified from UTEX 250-A genomic DNA. For the mCherry ER reporter (pJL105, Table 1), a DNA sequence encoding 1–39 aa of BIP1 (UTEX 250-A_A01.00955.1) was amplified from UTEX 250-A cDNA and assembled with the codon-optimized *mCherry* sequence ^76^ and a 12-bp extension for the HDEL retention sequence. The 1158-bp *LPAAT2* (UTEX 250-A_B12.35270.1) coding sequence was amplified from UTEX 250-A cDNA and fused with the codon-optimized *Venus* DNA sequence ^76^.

### Lipid extraction and fatty acid profiling.

The complete procedure is described in ^77^. Total lipids from lyophilized or fresh cell pellets obtained from 1–2 mL cell cultures were extracted using a modified Bligh and Dyer method, with chloroform replaced by dichloromethane ^78–80^. For fatty acid (FA) composition analysis, C13:0 (Sigma Aldrich 91988, 5 mg/mL in dichloromethane) was added to the lipid extracts as an internal standard. The total FAs were derivatized by incubating in 1N methanolic hydrochloric acid at 80 °C for 30 min to generate fatty acid methyl esters (FAMEs), dissolved in hexane for GC-MS analysis (Agilent 7890A GC system connected with MSD 5977A mass spectrometer, J&W DB-FastFAME GC column 60 m, 0.25 mm, 0.25 μm). Data obtained from GC-MS were analyzed using Agilent MassHunter Workstation Software (Quantitative Analysis ver. B.07.00 and Quantitative Analysis ver. B.06.00). Peaks of individual FAs were verified by comparing their retention time (RT) with FAMEs from standard mixes (Supelco CRM47885, Cayman Chemical 29365), targeted detection of *m*/*z* with extracted ion chromatogram (EIC), and spectrum search in the NIST (National Institute of Standards and Technology Library). The resulting data (integrated peak areas) were calibrated with standard curves (peak concentration) derived from external FAME standards. Data processing, statistical analysis, and plotting were performed using R 4.2.2.

### Lipase-mediated regiochemical analysis of TAG.

The complete procedure, adapted from Luddy et al., 1964 ^81^, is described in ^77^. Briefly, 4.5–5 mg of extracted total lipids were digested with 4 mg of *sn*-1, 3-specific porcine pancreas lipase (Sigma Aldrich L3126) in the presence of 0.018% taurocholic acid (Sigma Aldrich T4009) and 1.63% CaCl_2_ in 1 M Tris-Cl at pH 8.0 at 40 °C for 10 m. The resulting 2-monoacylglycerol (2-MAG) was separated on a thin-layer chromatography (TLC) Silica gel 60 plate (Sigma Aldrich 1057210001) in 101 mL of hexane: ethyl acetate: acetic acid = 50:50:1 (v/v) developing solvent. ^82,83^. The separated neutral lipids were visualized with 0.05% premuline (Sigma Aldrich 206865), and the 2-MAG fraction was collected from the TLC plate and extracted with dichloromethane, followed by methyl esterification for GC-MS analysis of the FA at the *sn*-2 position.

### Fluorescence microscopy of LPAAT proteins and ER structure.

Engineered strains cultured under lipid-induction conditions were collected on d5 for microscopy. Cells were embedded in 2% low-melting-point agarose and 150 mM sorbitol on the slide to minimize cell movement and to position the cells within the same focal plane. Fluorescent signals of target proteins within the cells were observed using a Zeiss LSM710 Confocal Microscope for multi-modal imaging of individual cells, utilizing single-channel confocal scanning of the individual fluorophores (mCherry: Ex/Em 561/596 nm, VENUS: Ex/Em 514/572 nm) and super-resolution imaging with the Airy Scan detector. Subsequent data processing used the Zeiss ZEN Blue 3.0 software.

### Tandem mass spectrometry analysis of triacylglycerols (TAGs). Standards and samples.

The following TAG isomer standards were purchased from ABITEC (Larodan, Monroe, MI): OPO (1,3-dioleoyl-2-palmitoyl glycerol; 34–1831-7), OOP (1,2-dioleoyl-3-palmitoyl glycerol; 34–1821-7), POP (1,3-palmitoyl-2-dioleoyl glycerol; 34–1611-7), PPO (1,2-palmitoyl-3-dioleoyl glycerol; 34–1601-7), OMO (1,3-olein-2-myristin; 34–1830-7), OOM (1,2-olein-3-myristin; 34–1820-7), OLaO (1,3-olein-2-laurin; 34–1819-7), OOLa (1,2-olein-3-laurin; 34–1817-7), OPL (1-olein-2-palmitin-3-linolein; 34–3035-7), PLO (1-palmitin-2-linolein-3-olein; 34–3010-7), POL (1-palmitin-2-olein-3-linolein; 34–3012-7), LaPO (1-laurin-2-palmitin-3-olein; 34–3039-7), POLa (1-laurin-2-olein-3-palmitin; 34–3004-7), and OLaP (1-olein-2-laurin-3-palmitin; 34–3046-7). Individual standards were prepared as 50 ng/μL solutions in 4:1 acetone:methanol (volume/volume) first and then into three sets of standard mixtures. The standard mixtures of Set 1 were made of solutions of 0, 1.5625, 3.125, 6.25, 12.5, and 25 ng/μL OPO, OMO, OLaO, POL, and POLa. The standard mixtures of Set 2 were pairs of OPO+OOP, POP+PPO, OMO+OOM, and OlaO+OOLa mixed in the ratios of 1:0 (100%), 1:1 (50%), and 3:1 (25%), at a total concentration of 25 ng/μL (5 mixtures for each isomer pair). Set 3 comprised individual OPL, PLO, POL, OPLa, PLaO, and POLa, each at a concentration of 25 ng/μL. For algal samples, the extracted total lipids were resuspended with 4:1 acetone:methanol to a final concentration of 50 ng/μL ^84^.

### Liquid chromatography-mass spectrometry and tandem mass spectrometry.

Lipid extract samples and TAG standards were analyzed using a liquid chromatography (LC) system (1200 Series, Agilent Technologies, Santa Clara, CA) that was connected in line with an LTQ-Orbitrap-XL mass spectrometer equipped with an electrospray ionization (ESI) source (Thermo Fisher Scientific, Waltham, MA). The instrumentation is located in the QB3 Mass Spectrometry Facility at the University of California, Berkeley. The LC system was equipped with a G1322A solvent degasser, G1311A quaternary pump, G1316A thermostatted column compartment, and G1329A autosampler unit (Agilent). The column compartment was equipped with a Zorbax 300SB-C18 column (length: 150 mm, inner diameter: 2.1 mm, particle size: 5 micrometers, part number 883750–902, Agilent). Ammonium formate (99%, Alfa Aesar, Ward Hill, MA), 2-propanol, methanol (Optima LC-MS grade, 99.9% minimum, Fisher, Pittsburgh, PA), and *n*-hexane (95% minimum, Thermo) were used to prepare mobile phase solvents. Mobile phase solvent A was methanol with 1 mM ammonium formate and mobile phase solvent B was 50% 2-propanol/50% *n*-hexane (volume/volume). The elution program consisted of isocratic flow at 2% (volume/volume) B for 2 min, a linear gradient to 20% B over 23 min, a linear gradient to 95% B over 2 min, isocratic flow at 95% B for 5 min, a linear gradient to 2% B over 2 min, and isocratic flow at 2% B for 26 min, at a flow rate of 300 μL/min. The column compartment was maintained at 25 °C and the sample injection volume was 1 μL. External mass calibration was performed using the Pierce LTQ ESI positive ion calibration solution (catalog number 88322, Thermo). Full-scan, high-resolution mass spectra were acquired in the positive ion mode over the range of mass-to-charge ratio (*m*/*z*) = 350 to 1000, using the Orbitrap mass analyzer, in profile format, with a mass resolution setting of 60,000 (at *m*/*z* = 400, measured at full width at half-maximum peak height, FWHM). Tandem mass (MS/MS or MS2) spectra of selected TAG precursor ions, [M+NH_4_]^+^, were acquired using collision-induced dissociation (CID) in the linear ion trap, in centroid format, with the following parameters: isolation width = 3 *m*/*z* units, normalized collision energy = 30%, activation Q = 0.25, and activation time = 30 ms. Preliminary data acquisition and analysis were performed using Xcalibur software (version 2.0.7, Thermo).

### Data analysis.

The raw data were processed with MZmine 4.7.29 for mass detection and targeted feature detection, and the resulting MS2 scans (.mgf) were loaded to R with Spectra 1.20.0 for further analysis ^85,86^. The stereostructure of a TAG molecule can be inferred from the relative abundance of daughter ions generated from the loss of a fatty acyl chain during fragmentation ^84^. In this process, the *sn*-1 and 3 acyl groups are more susceptible to cleavage, leading to a higher ratio of *sn*-1,2 (or *sn*-2,3) to *sn*-1,3 diacylglycerols. For example, the POP precursor generates daughter ions of PO (*m*/*z* 577.5) and PP (*m*/*z* 551.5) at a ratio (PO/PP) of 4.0, while the same ratio yielded from PPO is only 1.2. Standard curves of the ratios from different mixing ratios of the two regioisomers in the standard set 2 above were generated using the nonlinear least squares model to estimate the fraction of each isomer in the algal samples based on the measured ratio of fragments. For isomers with three different acyl groups like OPL, PLO, and POL, the fraction of the three resulting fragments is determined by the fraction of each of the three isomers as follows:

$$C_{PL1}X+C_{PL2}Y+C_{PL3}Z=F_{PL}$$

$$C_{PO1}X+C_{PO2}Y+C_{PO3}Z=F_{PO}$$

$$C_{OL1}X+C_{OL2}Y+C_{OL3}Z=F_{OL}$$,

where C represents the coefficient of each fragment (PL, PO, OL), F is the resulting fraction of each fragment which is correlated to the percentage of isomer OPL (X), PLO (Y), and POL (Z). The coefficients were obtained from the fragmental fraction measured in each isomer standard (set 3), and the subsequent coefficient matrix was used for non-negative least squares modeling to calculate the values of X,Y, and Z in algal samples (R package lsei 1.3) ^87^.

We noticed the presence of chimeric MS2 spectra from some TAG precursors in algal samples, which was caused by co-elution of isobaric TAGs. For example, SMO (or SOM and OSM) shares the same precursor ion *m*/*z* with POP and PPO, which can be identified from the two additional fragment ions at *m*/*z* 549.5 (MO) and *m*/*z* 605.5 (SO) in the MS2 spectrum. However, the third fragment ion, SM, overlaps with the PP fragment ion of POP/PPO at *m*/*z* 551.5. To calculate the proportion of the fragment ion abundance at *m*/*z* 551.5 derived solely from PP, we used the following equation, assuming that the fraction of the SM fragment ion abundance (X) at *m*/*z* 551.5 corresponds to the fraction of the sum of fragment ion abundances (I) from SMO/SOM/OSM relative to the total from both SMO/SOM/OSM and POP/PPO:

$$\frac{X}{I_{PP+SM}}=\frac{I_{MO}\;+\;I_{SO}\;+\;X}{I_{MO}\;+\;I_{PP+SM}\;+\;I_{PO}\;+\;I_{SO}}$$

In addition, a target-specific RT window of ±0.125 min was applied to filter the MS2 spectra for downstream analysis, which was particularly useful in separating MS2 spectra of target TAG species from MS2 spectra of non-target TAG species with slightly different RT values (e.g., a +0.375 min RT difference for SML/SLM/LSM compared to OMO/OOM). Finally, we calculated the sum of the calibrated peak intensities to quantify TAGs using the corresponding standard curves generated from standard set 1 with the four-parameter logistic model (R package drc 3.0) ^88^.

### Sample preparation for proteomics.

Cell pellets were resuspended in 8 M urea and transferred to 2 mL prefilled Micro-Organism Lysing Mix glass bead tubes. Samples were homogenized using a Bead Ruptor Elite bead mill homogenizer (OMNI International, Kennesaw, GA, USA) at speed 5.5 for 45 s. Immediately after bead beating, lysates were placed on an ice block and then centrifuged into 4 mL tubes at 1,000 × g for 10 min at 4 °C. Protein concentrations were determined using a bicinchoninic acid (BCA) assay according to the manufacturer’s instructions (Thermo Scientific, Waltham, MA, USA). For protein reduction, dithiothreitol (DTT) was added to a final concentration of 10 mM, and samples were incubated at 60 °C for 30 min with constant shaking at 800 rpm. Iodoacetamide (IAA) was then added to a final concentration of 40 mM, and samples were incubated for 45 min at room temperature in the dark. Samples were diluted to 4 M urea with 100 mM NH_4_HCO_3_ and 1 mM CaCl_2_, followed by digestion with Lys-C (Fujifilm Wako, Richmond, VA, USA) at a 1:20 enzyme-to-protein ratio (w/w) for 3 h at 37 °C. Samples were then further diluted to 1 M urea and digested with sequencing-grade modified trypsin (Promega, Madison, WI, USA) at a 1:50 enzyme-to-protein ratio (w/w) for 12 h at 37 °C with 800 rpm shaking. Digested peptides were desalted using a 4-probe positive-pressure Gilson GX-274 ASPEC^™^ system (Gilson Inc., Middleton, WI, USA) equipped with Discovery C18 50 mg/1 mL solid-phase extraction tubes (Supelco, St. Louis, MO, USA). Columns were conditioned with 3 mL methanol and equilibrated with 3 mL 0.1% trifluoroacetic acid (TFA) in H_2_O. Samples were loaded onto the columns and washed with 4 mL 95:5 H_2_O:acetonitrile (ACN) containing 0.1% TFA. Peptides were eluted with 1 mL 80:20 ACN:H_2_O containing 0.1% TFA. Eluted peptides were concentrated to approximately 100 μL using a SpeedVac concentrator. Peptide concentrations were determined by Bicinchoninic Acid (BCA )assay, and samples were diluted to 0.10 μg/μL with nanopure water prior to proteomics LC-MS/MS analysis.

### LC-MS/MS of peptides.

A Waters nano-Acquity dual pumping Ultra Performance Liquid Chromatography system (Milford, MA) was configured for on-line trapping of a 5 μL injection at 5 μL/min for 10 min followed by reverse-flow elution onto the analytical column at 200 nL/min. The trapping column was slurry packed in-house using 360 μm outside diameter (o.d.) × 150 μm inside diameter (i.d.) fused silica (Polymicro Technologies Inc., Phoenix, AZ) Jupiter 5μm octadecylsilane C18 media (Phenomenex, Torrence, CA) with 2 mm sol-gel frits on either end. The analytical column was slurry packed in-house using Waters BEH 1.7 μm particles packed into a 35 cm long, 360 μm o.d. × 75 μm i.d. column with an integrated emitter (New Objective, Inc., Littleton, MA). Mobile phases consisted of (A) 0.1% formic acid in water and (B) 0.1% formic acid in acetonitrile with the following gradient profile (min, %B): 0, 1; 10, 8; 105, 25; 115, 35; 120, 75; 123, 95; 129, 95; 130, 50; 132, 95; 138, 95; 140,1. MS analysis was performed using an Thermo Eclipse mass spectrometer (Thermo Scientific, San Jose, CA). The ion transfer tube temperature and spray voltage were 300°C and 2.4 kV, respectively. Data were collected for 180 min following a 27 min delay from when the trapping column was switched in line with the analytical column. High-Field Asymmetric Waveform Ion Mobility Spectrometry (FAIMS) was used at compensation voltages −40V, −60V, and −80V. Fourier Transform Mass Spectrometry (FT-MS) spectra were acquired from 300–1800 m/z at a resolution of 120k (automatic gain control (AGC) target 4 × 10^5^) and while the top 12 Fourier Transform Higher-energy Collisional Dissociation (FT-HCD)-MS/MS spectra were acquired in data dependent mode with an isolation window of 0.7 m/z and at an orbitrap resolution of 50K (AGC target 5 × 10^4^) using a fixed collision energy (HCD) of 32% and a 30 s exclusion time.

### Database Searching and Sequence Identification.

Raw LC-MS/MS files were searched using the MSGFPlus algorithm ^89^ against the *Auxenochlorella* UTEX 250-A v1.1 database (16,930 entries) ^36^ supplemented with a library of commonly observed contaminant proteins (Trypsin, Keratins, etc; 16 entries). Search parameters included a parent mass tolerance of ±20 ppm and dynamic oxidation of methionine as a variable modification. A target/decoy approach was employed to assess the False Discovery Rate (FDR). Precursor ion abundances were extracted from Selected Ion Chromatograms (SICs) using MASIC software, specifically calculating the “StatMomentsArea” (area under the elution curve) ^90^. Data were collated using MAGE Extractor and File Processor (https://github.com/PNNL-Comp-Mass-Spec/Mage) and managed within a centralized SQL Server database. Resulting Peptide-to-Spectrum Matches (PSMs) were filtered to a 1% FDR (Q-value ≤ 0.01029 yielding 8.923 decoy PSMs out of 892,326 total filter passing) to generate a high-confidence list of peptide identifications per sample.

### Protein Inference and Parsimony Mapping.

Clean peptide sequences sequences (pre- and suffix amino acids as well as modification callouts) were mapped back to the search FASTA using Protein Coverage Summarizer. To resolve the degeneracy of peptide-to-protein relationships, a hierarchical parsimony approach was implemented to derive a “BestGeneID” for each sequence: 1) Unique Mapping: If a sequence mapped to only one GeneID, it was assigned as the BestGeneID. 2) Unique Sequence Preference: If multiple GeneIDs were associated, but only one possessed a unique clean sequence elsewhere, that GeneID was selected. 3) Frequency of Observation: If neither condition was met, the GeneID with the highest count of other associated clean sequences was chosen. 4) Database Index: In cases of remaining ties, the GeneID appearing first in the indexed FASTA file was assigned. Highly non-unique sequences (mapping to multiple GeneIDs with unique sequences) were excluded from further quantitation (0.61% of data).

### Data Normalization and Statistical Assessment.

Bioinformatic processing was performed using Inferno (a .NET wrapper for R) ^91^. Summed peptide abundances were log_2_ transformed and normalized using a mean central tendency approach. Global data quality was assessed via box-plots, Pearson correlation heatmaps, and 2D/3D Principal Component Analysis (PCA). For protein-level quantification, normalized peptide abundances were de-logged, summed according to their assigned BestGeneID, and re-transformed to log_2_ values. Differential expression was assessed via pairwise comparisons of biological replicates (n=4). Proteins required observation in at least two replicates to pass the averaging step. A Limit of Quantitation (LOQ) was defined as two standard deviations below the mean of all averaged abundances. Statistical significance was determined using a log_2_ ratio distribution-based T-test (Z-score). GeneIDs were considered significantly differentially expressed if they met the following criteria: a |Z-score| > 2, a corresponding *p*-value < 0.05, and identification based on more than one unique peptide. Results were categorized into 18 assessment tiers to facilitate comparative analysis across all experimental conditions.

## Supplementary Material

Supplement 1

Supplement 2

## Figures and Tables

**Table 1.** Regiospecific Distribution of Palmitate, MCFA, and Linoleate

	HMF	*P. moriformis* ^c^	*A. thaliana* ^d^	*Y. lipolytica* ^e^	*Auxenochlorella* UTEX 250-A^f^						
					Wild type	JL27	JL29	JL52 +JL27	JL88 +JL27	JL88 +JL73	JL116 +JL88 +JL27
n	n/a	n/a	n/a	n/a	**6**	**4**	**4**	**3**	**3**	**4**	**4**
C16:0% total^ab^	25.0	31.5	~23.0	~24.0	10.00±0.04	11.0±0.9	10.7±0.7	13.7±0.7	20.1±0.6	20.60±0.14	20.7±0.8
C16:0% *sn*-2^ab^	51.0	69.3	~58.0	~50.0^e^	1.0±0.5	25±2	23±2	36±2	43±2	42±3	45.2±0.9
*sn*-2% 16:0^ab^	70.0–88.0	73.3	~84.0	69.1	3.2±1.6	75.5±0.9	72±2	86.4±1.2	71±2	68±5	73±4
MCFA^†^% total^ghi^	13.4–28.6	n/a	n/a	n/a	1.01±0.03	1.27±0.10	1.22±0.08	29±1	21±2	22.7±0.4	24±2
MCFA% *sn*-2^gh^	14.8–19.5	n/a	n/a	n/a	0.21±0.04	0.91±0.09	1.46±0.03	13.7±0.8	15.7±0.4	18.3±0.5	19±3
*sn*-2% MCFA^gh^	35.5–49.3	n/a	n/a	n/a	7±2	24.0±0.9	40±2	15.9±1.6	25±2	27±1	28±4
C18:2% total^aj^	13.0–21.4	n/a	n/a	n/a	9.90±0.04	13±2	13.4±1.7	10.0±0.7	11.1±0.6	9.1±0.3	25.4±1.1
C18:2% *sn*-2^aj^	8.40–10.8	n/a	n/a	n/a	11.50±0.16	9±2	14±2	7.8±0.3	8.3±0.5	6.4±0.8	12.3±0.4
*sn*-2% 18:2^aj^	16.8–21.5	n/a	n/a	n/a	38.7±0.6	23±2	34.4±0.9	26.2±1.1	25.00±0.17	23±3	16±1

a Innis. *Adv Nutr. 2 (2011)* ^3^

b Hageman et al. *Int. Dairy J. 92 (2019)* ^58^

c Zhou et al. *Front. Nutr. 11 (2024)* ^34^

d van Erp et al. *Metab. Eng. 67 (2021)* ^28^

e Bhutada et al. *Metab. Eng. Commun. 14 (2022)* ^31^

f This study

g Calculated from López-López et al. *Eur. J. Clin. Nutr. 56 (2002)* ^94^

h Calculated from Sun et al. *Food Chemistry 242 (2018)* ^95^

i Yuhas et al. *Lipids 41 (2006)* ^10^

j He et al. *Food Sci Nutr. 12 (2021)* ^96^

† C10:0-C14:0
