# Supplementary material for: Bioengineered algal lipids enriched in structured medium- and long-chain triacylglycerols, linoleate, and sn-2 palmitate for human milk fat substitutes
Source: bioRxiv. 2026 May 16:2026.05.15.725531. Preprint. [Version 1] doi: 10.64898/2026.05.15.725531 (PMC13192765; doi:10.64898/2026.05.15.725531)
Supplement: Supplement 1 [file media-1.pdf]

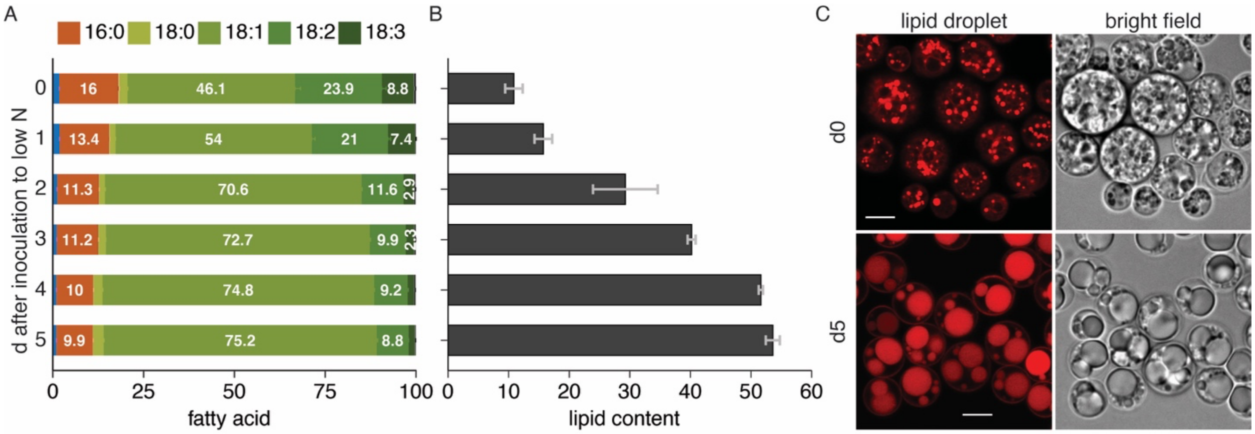

Figure S1. Lipid accumulation in *Auxenochlorella* cells. Changes in **(A)** fatty acid composition (% of total FA), **(B)** lipid content (% per dry weight), and **(C)** Lipid droplet morphology over a 5-day growth period of heterotrophic cell culture after transfer to low-nitrogen (3.75 mM NH_4^+) medium. Refer to Materials and Methods for detailed growth conditions of cultures and microscopy. Staining cells on day 0 and 5 with LipidTOX™ and observed using confocal microscopy. Scale bar = 5 μm.

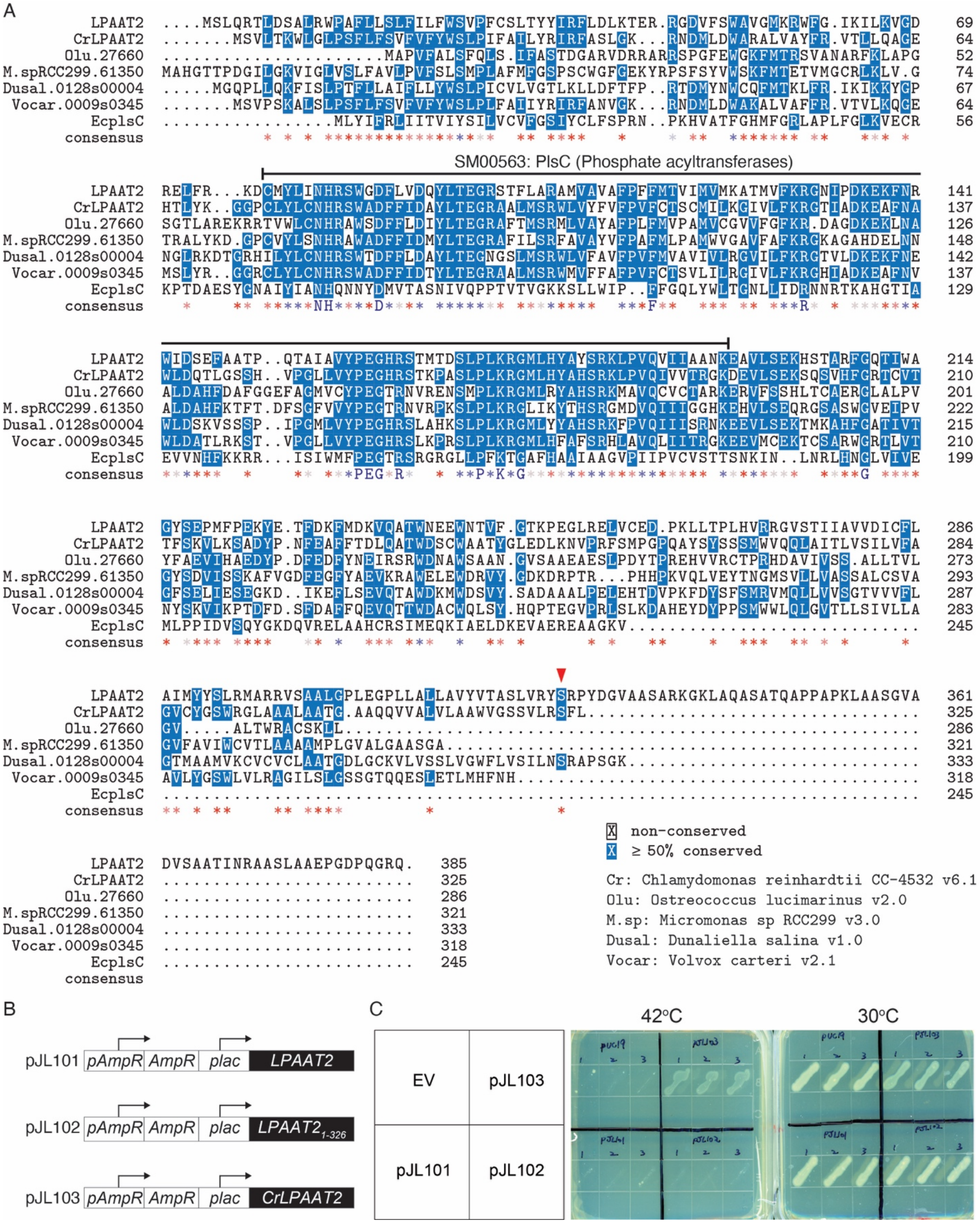

A
SM00563: PlsC (Phosphate acyltransferases)
LPAAT2
CrLPAAT2
Olu.27660
M.spRCC299.61350
Dusal.0128s00004
Vocar.0009s0345
EcplsC
consensus
X non-conserved
X ≥ 50% conserved
Cr: Chlamydomonas reinhardtii CC-4532 v6.1
Olu: Ostreococcus lucimarinus v2.0
M.sp: Micromonas sp RCC299 v3.0
Dusal: Dunaliella salina v1.0
Vocar: Volvox carteri v2.1
B
pJL101 pAmpR AmpR plac LPAAT2
pJL102 pAmpR AmpR plac LPAAT2 1-326
pJL103 pAmpR AmpR plac CrLPAAT2
C
42°C
30°C
EV
pJL103
pJL101
pJL102

Figure S2. Sequence and functional characterization of LPAAT2 in UTEX 250-A.

(A) Multiple sequence alignment of the putative LPAAT2 in UTEX 250-A and LPAAT/AGPAT orthologs from other Chlorophyta and PlsC from *E. coli*. The predicted functional domain SM00563 (PlsC) / IPR002123 (Phospholipid/glycerol acyltransferase) of LPAAT2 is indicated. Conserved residues are marked with '＊', which is scaled from red (low) to blue (high) based on the conservation score. Consensus residues are marked with uppercase letters. Red triangle indicates the S326 residue of LPAAT2. The peptide sequences were obtained from Phytozome 14 (Chlorophyte orthologs) and UniProt (EcPlsC), and the sequence alignment was performed using the CLUSTAL 2.1 algorithm with the msa 1.42 package [12]. **(B)** LPAAT2 and CrLPAAT2 expression constructs for complementation test of the *E. coli plsc* mutant. The sequences have been codon-optimized for expression in *E. coli.* pJL102 contains the truncated LPAAT2 with deletion of the 327-385 region. **(C)** The expression vectors, including the empty vector (EV, pUC19), were transformed into the *E. coli* plsc mutant (strain ID: SMC2-1) and grown at 30°C or 42°C.

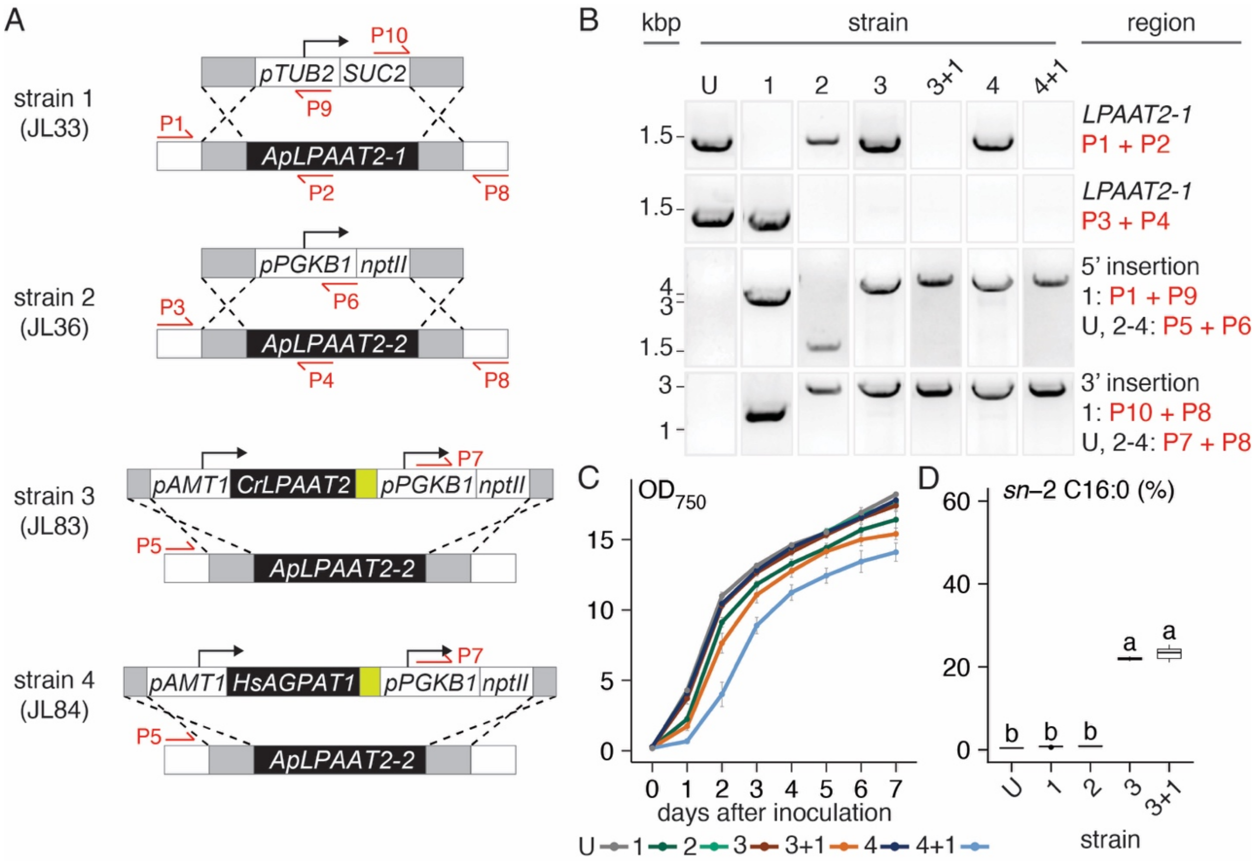

Figure S3. Heterologous expression of LPAAT2 in *lpaat2* mutants of UTEX 250-A. (A) Schematic of the constructs and landing sites. Yellow bricks represent *VENUS.* Primers designed for PCR-genotyping the resulting strains are indicated by red arrows (Table S3). The endogenous *LPAAT2* alleles 1 and 2 are disrupted in *lpaat2-1*(JL33; strain 1) and *lpaat2-2* (JL36; strain 2), respectively. The CrLPAAT2-VENUS construct (JL83) is introduced into the *LPAAT2-2* locus in UTEX 250-A and *lpaat2-1* (strain 1) to generate *CrLPAAT2-VENUS::lpaat2-2* (strains 3) and *CrLPAAT2-VENUS::lpaat2* (3+1), respectively. **(B)** PCR-genotyping of strains 1-3 to verify the disruptions in endogenous *LPAAT2* and *CrLPAAT*. **(C)** Growth curves of strain 1-4 and UTEX 250-A under low-N conditions over a 7-day course. Day 0 = the day of inoculation to the low-N medium. **(D)** 16:0 content (% of total FA) measured from 2-MAG. Letters represent Tukey Honest

Significant Differences between strains (*padj* ≥0.05 for measurements with the same letter). n = 3-4. Refer to Table S1. for the strain details.

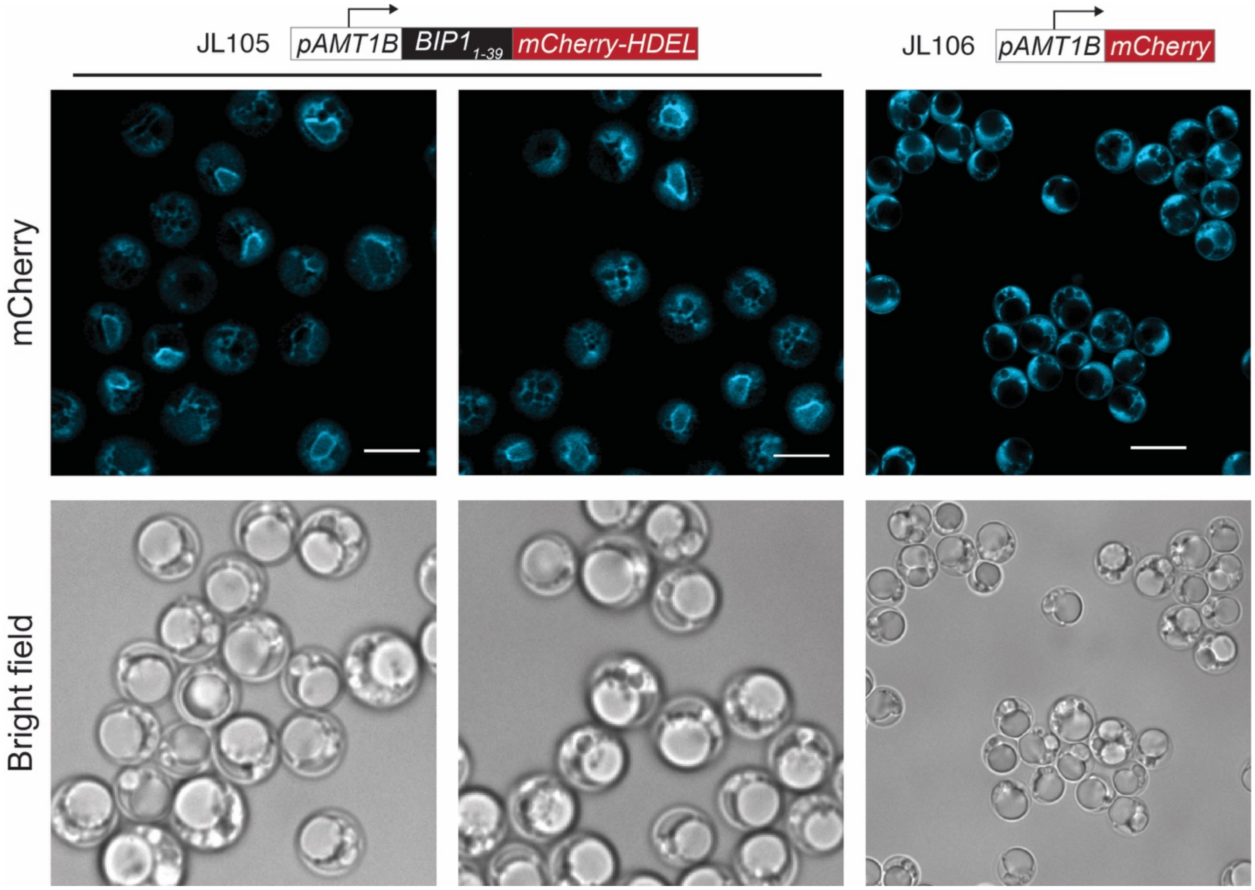

Figure S4. ER-localization of mCherry fluorescent reporter. Fluorescence confocal microscopy of ER-signal peptide-tagged mCherry-HDEL marker (JL105) and mCherry-only control (JL106). Scale bar = 5 μm. Cells were grown in the lipid production phase for 5 days.

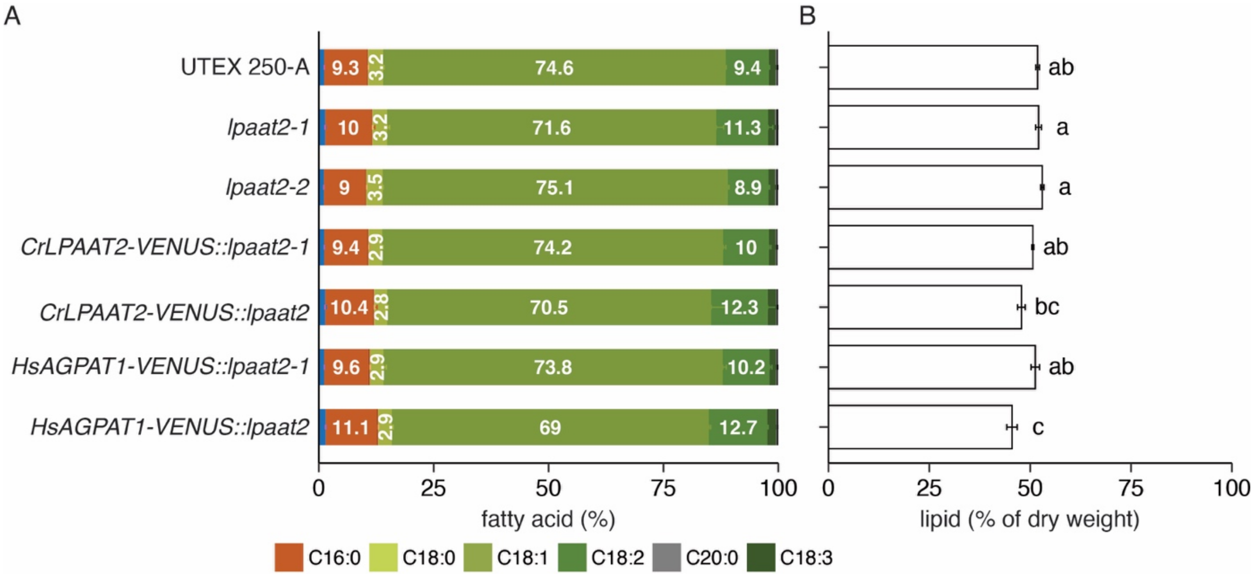

Figure S5. Lipid accumulation in *lpaat2* complementation strains. (A) fatty acid composition (% of total FA) and **(B)** lipid content (% per dry weight) of *CrLPAAT-VENUS* and *HsAGPAT1*-expressing strains and their respective controls from day-5 cell cultures in Fig. 5. Letters represent Tukey Honest Significant Differences between strains (*padj* ≥0.05 for measurements with the same letter). n = 3-4. Refer to Table S1. for the strain details.

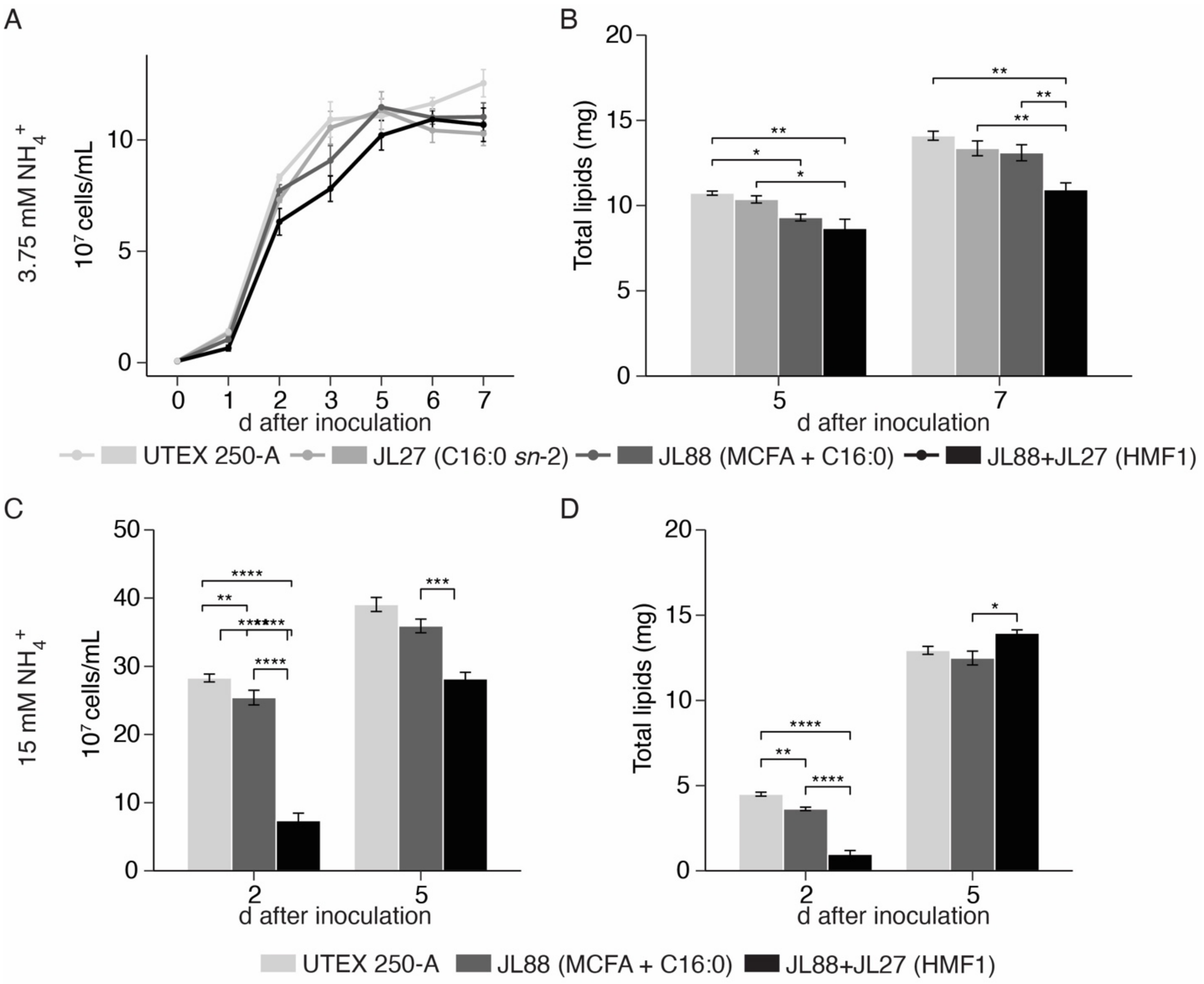

Figure S6. Cell growth and lipid yield of HMF-producing UTEX 250-A strains. (A) Cell density measurements of the wild type (UTEX 250-A) and engineered strains of *CrLPAAT2* (JL27), *CwFATB2* + *BjFATB3* in the wild-type (JL88) and JL27 (JL88+JL27) backgrounds in low nitrogen medium. **(B)** Measurements of total lipids (mg) from 2 mL of cell cultures in A. Error bars = SEM. n = 3 independent transformations for each engineered strain and n=3 independent cultures of the wild type. **(C)** Cell density measurements of the UTEX 250-A and transgenic strains of *CwFATB2* + *BjFATB3* in the wild-type (JL88) and JL27 (JL88+JL27) backgrounds in nitrogen-replete medium. **(D)**

Measurements of total lipid yield (mg) extracted from 2 mL of cell cultures shown in part C. Error bars = SEM. n = 6 (JL88) and 4 (JL88+JL27) independent transformations for engineered strains and n=3 independent cultures of the wild type. Statistical significance was determined using a pairwise two-sample t-test (ns: $P \geq 0.05$, *$P < 0.05$, **$P < 0.01$, ***$P < 0.001$, ****$P < 0.0001$).

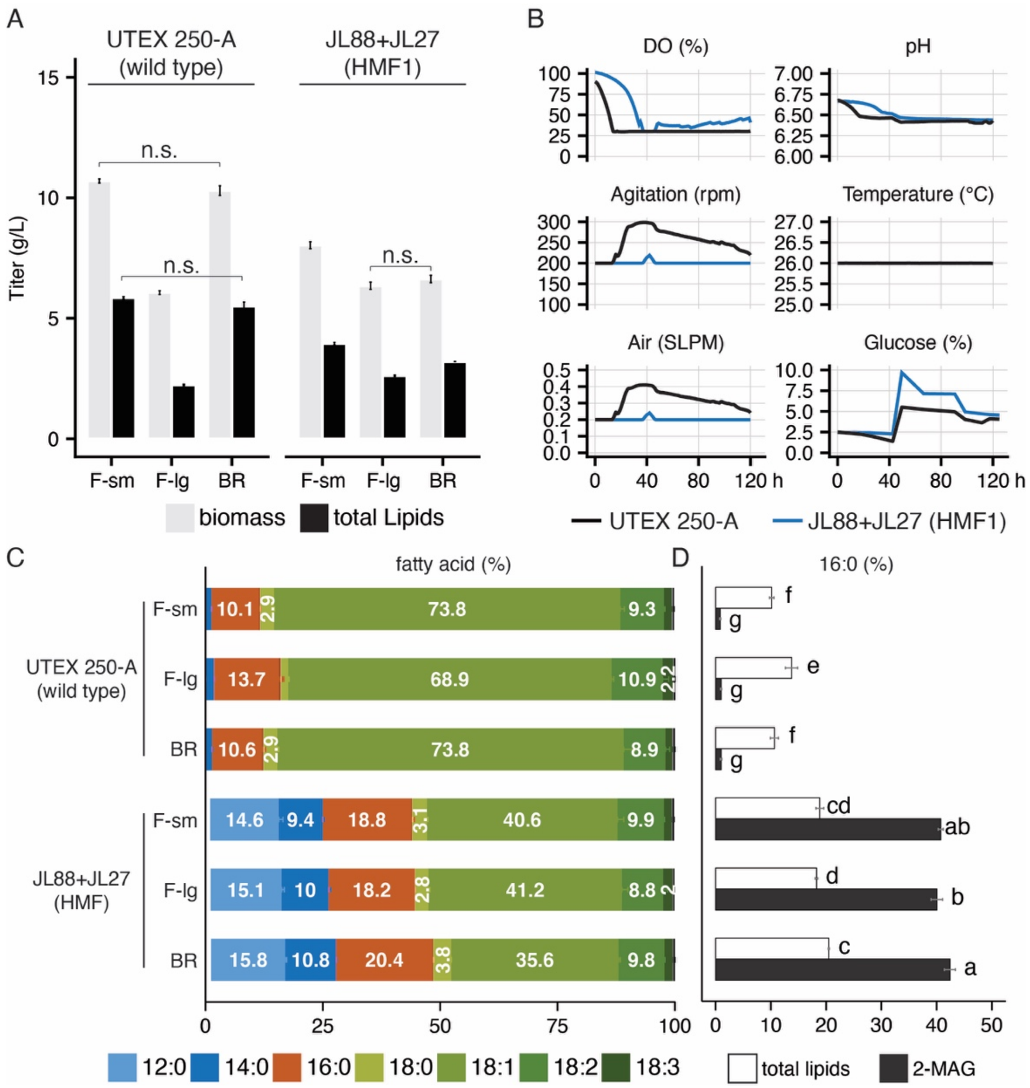

Figure S7. Scaling up cultivation of HMF-engineered strain for lipid production. The engineered strain JL88+JL27 and wild-type control UTEX 250-A were inoculated in low-N ApM1 medium at identical inoculation ratios (v/v) in three vessel types: small flask (F-sm, 25 mL culture volume, 125 mL capacity), large flask (F-lg, 2 L culture volume, 2.8 L capacity), and bioreactor (BR, 2 L culture volume, 3 L capacity), as described in Materials and Methods. **(A)** Biomass and lipid titers (g/L) measurements of the day-5 cell cultures of different vesicle types **(B)** Bioreactor data recorded over a 5d (120 h) course in the low-N medium. DO%: dissolved O_2, Air (Standard Litres Per Minute or SLPM): 2.5% (w/v)

glucose was provided on d 0 and gradually increased to 4% (UTEX 250-A) and 9.7% (JL88+JL27). **(C and D)** FA composition and 16:0 content measured from total lipids (white) and 2-MAG (black).

Strain	Transgene	Anticipated activity	Marker gene	Insertion site	Line #
UTEX 250-A	NA, control	NA	NA	none	NA
JLM63	NA, control	increased 18:0	*nptII*	*fab2A-2**	3
JLM82	*CwFATB2*	MCFA (10:0-14:0)	*nptII*	*fab2A-2*	3
JL15	*FAB2Tp-CwFATB2*	MCFA (10:0-14:0)	*nptII*	*fab2A-2*	3
JLM24	NA, control	NA	*nptII*	*dao1*†	4
JL20	*CwFATB2*	MCFA (10:0-14:0)	*nptII*	*dao1*	4
JL21	*FAB2Tp-CwFATB2*	MCFA (10:0-14:0)	*nptII*	*dao1*	3
JL13	*BjFATB3*	16:0	*nptII*	*fab2A-2*	4
JL86	*FAB2Tp-BjFATB3*	16:0	*nptII*	*fab2A-2*	3
JL44	*BjFATB3*	16:0	*nptII*	*dao1*	4
JL60	*FAB2Tp-BjFATB3*	16:0	*nptII*	*dao1*	5
JL27	*CrLPAAT2*	*sn*-2 16:0	*SUC2*	*dao1*	4
JL29	*HsAGPAT1*	*sn*-2 16:0	*SUC2*	*dao1*	4
JL52	*FAB2Tp-CwFATB2*<br>*BjFATB3*	MCFA (10:0-14:0)<br>16:0	*nptII*	*amt1B-1*◇<br>*dao1*	4
JL52<br>+JL27	*FAB2Tp-CwFATB2*<br>*BjFATB3*<br>*CrLPAAT2*	MCFA (10:0-14:0)<br>16:0<br>*sn*-2 16:0	*nptII*<br>*SUC2*	*amt1B-1*<br>*dao1*	3
JL88	*FAB2Tp-CwFATB2*<br>*FAB2Tp-BjFATB3*	MCFA (10:0-14:0)<br>16:0	*nptII*	*amt1B-1*<br>*dao1*	3
JL88<br>+JL27	*FAB2Tp-CwFATB2*<br>*FAB2Tp-BjFATB3*<br>*CrLPAAT2*	MCFA (10:0-14:0)<br>16:0<br>*sn*-2 16:0	*nptII*<br>*SUC2*	*amt1B-1*<br>*dao1*	3
JL88<br>+JL73	*FAB2Tp-CwFATB2*<br>*FAB2Tp-BjFATB3*<br>*CrLPAAT2*	MCFA (10:0-14:0)<br>16:0<br>*sn*-2 16:0	*nptII*<br>*SUC2*	*amt1B-1*<br>*lpaat2-1*	4
JL116	*AtLPCAT1*	18:2 (n-6c)	*ptxD*	*thi4*†	3
JL116<br>+JL88<br>+JL27	*AtLPCAT1*<br>*FAB2Tp-CwFATB2*<br>*FAB2Tp-BjFATB3*<br>*CrLPAAT2*	18:2 (n-6c)<br>MCFA (10:0-14:0)<br>16:0<br>*sn*-2 16:0	*ptxD*<br>*nptII*<br>*SUC2*	*thi4*<br>*amt1B-1*<br>*dao1*	4
JL117	*AtPDCT1*	18:2 (n-6c)	*ptxD*	*thi4*	3
JL117<br>+JL88<br>+JL27	*AtLPDCT1*<br>*FAB2Tp-CwFATB2*<br>*FAB2Tp-BjFATB3*<br>*CrLPAAT2*	18:2 (n-6c)<br>MCFA (10:0-14:0)<br>16:0<br>*sn*-2 16:0	*ptxD*<br>*nptII*<br>*SUC2*	*thi4*<br>*amt1B-1*<br>*dao1*	4

JL105	*ApBIP1*$_{1-39}$*-mCherry-HDEL*	ER marker	*SUC2*	*dao1*	3
JL106	*mCherry*	control	*SUC2*	*dao1*	3
JL36	NA, control	*lpaat2-2* knock-out	*nptII*	*lpaat2-2*	4
JL33	NA, control	*lpaat2-1* knock-out	*SUC2*	*lpaat2-1*	4
JL83	*CrLPAAT2-VENUS*	*sn*-2 16:0	*nptII*	*lpaat2-2*	4
JL84	*HsAGPAT1-VENUS*	*sn*-2 16:0	*nptII*	*lpaat2-2*	3
JL83+JL33	*CrLPAAT2-VENUS*	*sn*-2 16:0<br>reduced *sn*-2 18:x	*nptII*<br>*SUC2*	*lpaat2*	8
JL84+JL33	*HsAGPAT1-VENUS*	*sn*-2 16:0<br>reduced *sn*-2 18:x	*nptII*<br>*SUC2*	*lpaat2*	4
JL105<br>+JL81	*ApBIP1*$_{1-39}$*-mCherry-HDEL*<br>*ApLPAAT2-VENUS*	ER marker<br>Fluorescence-labeled ApLPAAT2 (putative)	*nptII*<br>*SUC2*	*lpaat2-2*<br>*dao1*	3
JL105<br>+JL83	*ApBIP1*$_{1-39}$*-mCherry-HDEL*<br>*CrLPAAT2-VENUS*	ER marker<br>Fluorescence-labeled CrLPAAT2	*nptII*<br>*SUC2*	*lpaat2-2*<br>*dao1*	3
JL105<br>+JL84	*ApBIP1*$_{1-39}$*-mCherry-HDEL*<br>*HsAGPAT1-VENUS*	ER marker<br>Fluorescence-labeled HsAGPAT1	*nptII*<br>*SUC2*	*lpaat2-2*<br>*dao1*	3

* increased 18:0 due to reduced stearyl-ACP desaturase activity
† heterozygous; neutral for growth and lipid production
◇ heterozygous; neutral for growth and lipid production due to its locus on a trisomic chromosome

Table S1. Strains generated in this study.

Name	Sequence	Description
P1	TACTGTACAGCAAAGCGATTCT	*LPAAT2-1* 5’ forward
P2	AGCTCCCCATTTTGACAAACACTTTTC	*LPAAT2-1* CDS reverse
P3	TACTGTACAGCAAAGCGATACT	*LPAAT2-2* 5’ forward
P4	TCCCCATGCTGACAAACGTTTTTT	*LPAAT2-2* CDS reverse
P5	CGTCCTCTTCAACACCATGTACT	*LPAAT2* 5’ forward (non-allele specific)
P6	GGAGGCCATTACACACGTCT	*pPGKB1* reverse
P7	TGGTGGGCAACATCATCCTC	*pPGKB1* forward
P8	AGTCGGTCAAGGGCTTCTTC	*LPAAT2* 3’ reverse (non-allele specific)
P9	TGGCTTTGGTAGCTCGCTATAAT	*pTUB2* reverse
P10	GCCTGCATGCACGTTTGTAT	*PGH* terminator forward

Table S4. DNA primers used in Fig. 5. Primers designed for genotyping *lpaat2* allelic mutants, as well as CrLPAAT2 and HsAGPAT1 insertions on both 5’ and 3’ ends of the insertion site.
